# Probing Structural Defects in MOFs Using Water Stability

**DOI:** 10.1021/acs.jpcc.3c07497

**Published:** 2024-02-23

**Authors:** Shubham Jamdade, Zhenzi Yu, Salah Eddine Boulfelfel, Xuqing Cai, Raghuram Thyagarajan, Hanjun Fang, David S. Sholl

**Affiliations:** † School of Chemical & Biomolecular Engineering, Georgia Institute of Technology, Atlanta, Georgia 30332-0100, United States; ‡ Oak Ridge National Laboratory, Oak Ridge, Tennessee 37830, United States

## Abstract

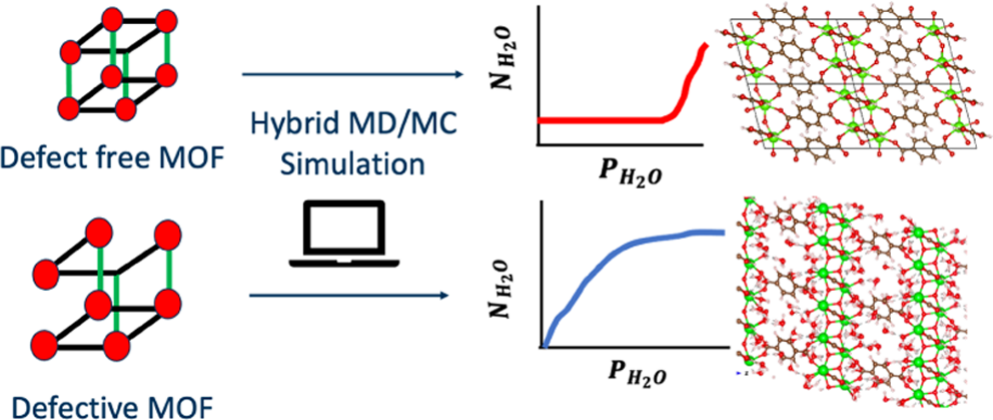

Defects in the crystal structures of metal–organic frameworks (MOFs), whether present intrinsically or introduced via so-called defect engineering, can play strong roles in the properties of MOFs for various applications. Unfortunately, direct experimental detection and characterization of defects in MOFs are very challenging. We show that in many cases, the differences between experimentally observed and computationally predicted water stabilities of MOFs can be used to deduce information on the presence of point defects in real materials. Most computational studies of MOFs consider these materials to be defect-free, and in many cases, the resulting structures are predicted to be hydrophobic. Systematic experimental studies, however, have shown that many MOFs are hydrophilic. We show that the existence of chemically plausible point defects can often account for this discrepancy and use this observation in combination with detailed molecular simulations to assess the impact of local defects and flexibility in a variety of MOFs for which defects had not been considered previously.

## 1 Introduction

Metal–organic frameworks (MOFs) are porous crystalline materials that are potential alternatives to traditional materials in applications, such as gas separation, storage, and catalysis. MOFs consist of inorganic metal ions or clusters connected to organic ligands through coordination bonds, forming highly porous crystalline structures. MOFs can be tuned to have a wide range of surface areas, pore sizes, and chemical functionality. Tens of thousands of distinct MOF crystal structures have been reported.^1−3^

Most of the literature on MOFs focuses on the ordered crystal structure of these materials; however, even the most carefully synthesized MOFs must contain a variety of defects.^4^ Defects can play an important role in controlling the adsorption, separation, and stability of MOFs. Defective MOFs typically possess larger pores and a greater surface area or pore volume, leading to increased adsorption. Wang et al. used computation to study the effect of missing linkers on isopropyl alcohol (IPA) adsorption and diffusion in UiO-66.^5^ Their results showed that missing linkers and the resulting larger accessible porous volume can lead to a larger adsorption capacity of IPA but that IPA binds more strongly with uncoordinated Zr in defective regions, which results in a much slower self-diffusion. Cai et al. performed DFT calculations to explore how defect formation associated with the presence of adsorbed water affects C_2_H_6_, C_3_H_8_, and n-C_4_H_10_ diffusion in Zn(tbip), a MOF that in its pristine form has 1D channels that would lead to single-file diffusion for these molecules.^6^ Gong et al. showed that similar effects control hydrocarbon diffusion in UTSA-280.^7^ Even small defect concentrations may have a potential impact on the properties or long-term stability of MOFs.^8^ Chen et al. observed that degradation of DMOF-1 by water is driven by water adsorption at defect sites in the MOF.^9^ In addition to intrinsic defects, defects can be deliberately included in MOFs during synthesis using what has come to be known as defect engineering.^10−14^ Islamov et al. showed that even a small concentration of linker vacancy defects in MOFs can have a significant impact on the thermal conductivity of MOFs and their observations suggested that the differences in the thermal conductivity values measured in experiments and estimated using computations can be attributed to the computational models not accounting for defects in the crystals.^15^

Despite the potential importance of defects in the properties of MOFs, direct experimental detection of defects in MOFs is challenging. For most of the tens of thousands of crystal structures that have been reported, no information at all is currently available on the existence or consequences of defects.

Several studies have used comparisons between experimental data and computational predictions for water adsorption to study the effect of defects in MOFs. The work by Chen et al. mentioned above used this approach to infer that water-driven degradation of DMOF-1 occurs because of defects in the MOF’s structure.^9^ Choi et al. using Monte Carlo and DFT calculations to examine the role of structural defects on water adsorption in MOF-801^16^ found that a high concentration of defects in the simulated structures was necessary to agree with experimental adsorption results. Ghosh et al. reported computational simulations of defects in UiO-66, showing that missing linker creates defect sites which makes UiO-66 more hydrophilic.^17^

Because of the importance of water in many applications, the stability of many MOFs to water exposure has been reported.^18,19^ Walton and co-workers assigned stability classifications to more than 200 MOFs that have been experimentally characterized after water exposure.^18^ These MOFs were broadly classified into four categories: (i) thermodynamically stable, (ii) high kinetic stability, (iii) low kinetic stability, and (iv) unstable. MOFs classified with low kinetic stability show some evidence of structural stability after exposure to water in the vapor phase but do not exhibit stability after exposure to high humidity conditions. MOFs that are classified as unstable show little structural stability after exposure to even small amounts of moisture in the vapor phase.^18^Table 1 lists some examples of low kinetic stability and unstable MOFs from the experimental classification mentioned above. Using this experimental classification, Batra et al. developed a machine-learning model to predict the water stability of additional MOFs.^20^ It is important to note that these classifications are based on experimental observations, so they reflect the properties of MOFs including the presence of any defects that exist under the reported synthesis and activation conditions and not the properties of idealized defect-free (pristine) materials.

**Table 1:** Examples of Low Kinetic Stability and Unstable MOFs from Experimental Observations

low kinetic stability MOFs		unstable MOFs	
common name	activated formula unit	common name	activated formula unit
Cu-BTC/HKUST	Cu_3_(BTC)_2_	IRMOF-1/MOF-5	Zn_4_O(BDC)_3_
Mg-MOF-74	Mg_2_(DOBDC)	MOF-177	Zn_4_O(BTB)_2_
Co-MOF-74	Co_2_(DOBDC)	MOF-508	Zn_2_(BDC)_2_BPY
MIL-101-NO2(Cr)	Cr_3_F(H_2_O)_2_O(BDC-NO_2_)_3_	UMCM-1	Zn_4_O(BDC)_3_(BTB)_4_
MIL-47-F(V)	V(O)(BDC-F)	UiO-BPY	Zr_6_O_6_(BPY)_12_
Ni-DMOF	Ni_2_(BDC)_2_(DABCO)	Zn-DMOF-NO_2_	Zn_2_(BDC-NO_2_)_2_(DABCO)
Zn-DMOF	Zn_2_(BDC)_2_(DABCO)	Zn-DMOF–OH	Zn_2_(BDC–OH)_2_(DABCO)
MIL-110 (Al)	Al_8_(OH)_12_(OH)_3_(H2O)_3_(BTC)_3_	Bio-MOF-11	Co_2_(AD)_2_(CH_3_CO_2_)
SIFSIX-3-Zn	Zn (PYR)_2_(SiF_6_)	MIL-47(V)	V(O)BDC
UiO-66-F	Zr_6_O_6_(BDC-F)_12_	MOF-505	Cu_2_(BPTC)

In this study, we show how the observed or predicted experimental water stability of MOFs can be used in many cases to infer the presence of point defects in real materials. This approach greatly expands the number of MOFs for which the presence of point defects can be deduced at the structural and mechanistic level. To achieve this goal, we first compare careful molecular simulations of defect-free (pristine) MOFs for structures that are known or predicted to have low kinetic stability or be unstable with respect to water from the work of Burtch et al.^18^ and Batra et al.^20^ A common, although not universal, outcome of these simulations is the prediction that the pristine material is hydrophobic. When this is the case, the prediction from these molecular simulations is in qualitative disagreement with experimental observations since hydrophobic materials can be expected to be stable upon exposure to water. We then perform additional simulations in which specific chemically plausible point defects in the MOF are created, and the impact of these defects on water adsorption is simulated. This approach frequently allows significant adsorption of water in the material, thus providing indirect evidence that the real material used experimentally contains similar defects. Our results significantly expand the number of MOFs for which information is available about the presence of defects under experimentally reported synthesis and activation conditions.

The hypothesis for the impact of water on MOF stability described above assumes that the effects of water occur inside the pores of a MOF. An alternative hypothesis is that the observed instability in the presence of water is driven by processes on the exterior surface of a MOF crystal, where the termination of building blocks and perhaps the hydrophilicity of the material can be quite different from the interior of a crystal. Even less is known about the detailed chemical structure of the external surfaces of MOFs than about the presence of point defects inside the MOF crystals, and we acknowledge that the simulations we report below cannot unambiguously rule out effects due to external crystal surfaces. We return to this issue in the Section 4, where we suggest experiments that may be useful for further resolving this situation.

## 2 Methods and Computational Details

### 2.1 MOF Structures

Combining the water stability data sets developed by Burtch et al.^18^ and Batra et al.,^20^ 89 MOFs are known or predicted to have low kinetic stability or be unstable upon exposure to water. Pristine MOF structures of these 89 MOFs were obtained from the CoRE MOF database^1^ or the CCDC database.^2^ Some of these MOF structures had stoichiometric discrepancies because of partial occupancies in the reported data, presence of free solvents, and/or missing hydrogen atoms. We cleaned a number of these structures manually, but this approach was unsuccessful in some cases due to structural complexity. Thus, we generated 35 computation-ready MOFs for detailed computational simulations of water adsorption. These structures are listed in Table S1.

Full cell geometry optimization was performed for all of these 35 MOFs with plane-wave DFT calculations using the Vienna Ab initio Simulation Package (VASP) with D3 dispersion corrections^21^ and the Perdew–Burke–Ernzerhof exchange–correlation functional.^22^ Geometry optimization using a conjugate gradient method and an energy cutoff of 600 eV was performed on pristine structures, with the relaxation of both lattice parameters and ionic positions until interionic forces reached less than 0.05 eV/Å. A 1 × 1 × 1 *k*-point mesh was used for all calculations. Atomic charges for the optimized geometry were assigned by the DDEC6 method. DDEC partial charges accurately reproduce the electrostatic potential in the MOF pores and hence provide an accurate representation of electrostatic interactions between the MOF and the adsorbates with polar and quadrupolar interactions.^23^Tables S2 and S3 contain the complete list of 35 MOFs with their structure–property data before and after full cell geometry optimization. Physical properties such as the pore size distribution were calculated using zeo++^24^ and surface area, void fraction and pore volume were estimated using iRASPA^25^ with nitrogen and helium as probe molecules, respectively (see Tables S2 and S3). Among these MOFs, changes in surface area and pore volume between −100 and +15% were observed upon optimization. Some MOFs show a distinct reduction in their surface area after optimization. In several cases, the pore size after optimization is smaller than the diameter (3.64 Å) of the probe, giving complete loss of the calculated surface area. We have not attempted to compare this observation to experimental data, although we note that there are many MOFs that are “nonporous” to N_2_ at 77 K because of kinetic effects but readily adsorb CO_2_ or similar molecules at ambient conditions. Optimized CIF files with atomic charges for each MOF are included in the Supporting Information.

### 2.2 Generation of Defective MOF Structures

There are at least three kinds of point defects known to exist in MOFs: (i) linker vacancies, (ii) metal center vacancies, and (iii) dangling linkers.^8^ Linker vacancies can be created by modulators or solvent molecules binding preferentially to metal sites instead of organic linkers.^4^ Reports of metal center vacancies are scarce.^4,10^ In a dangling linker defect, the “bridging” linker is bound to fewer metal cations than would be expected in a pristine structure. This defect can also be considered an intermediate in the pathway to a linker vacancy.^8^ Zhang et al. used DFT to characterize several kinds of point defects in ZIF-8, concluding that linker vacancy defects are more likely to exist relative to metal center vacancies and dangling linker defects.^8^ Computational results have suggested that extended defects can also exist in at least some MOFs.^26^

**Figure 1:**
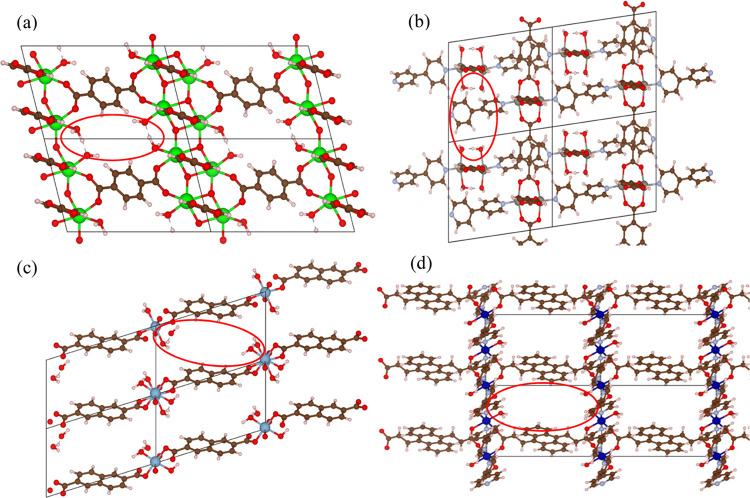
MOF supercells containing one missing linker per unit cell for (a) ZONBAH, (b) ATOXAJ, (c) PARPII, and (d) WONYEG. Red circles highlight the missing linker.

In this work, we studied the influence of linker vacancy and dangling linker defects. For each MOF of interest, we generated defective MOF structures by introducing low, moderate, and high concentrations of missing linker defects. We recently described robust methods for this task that can be applied to large collections of MOF crystal structures.^27^ There is evidence in ZIF materials that it is energetically favorable for point defects to be clustered,^28,29^ but we did not consider the effect of different spatial configurations for defects inside our simulated structures. We defined the varying defect density in terms of the defect concentration. The defect concentration is defined as the molar ratio of the missing linker or dangling linker; for example, one missing or dangling linker out of 10 linkers in a structure defines a defect concentration of 0.1. For low defect concentrations, we removed one linker from the 2 × 2 × 2 supercell, for moderate defect concentrations one linker was removed from a 1 × 2 × 1 supercell, and for high defect concentrations we removed one linker per unit cell. The specific defect concentrations associated with these three situations for each MOF are listed in Table S4. Similarly for some MOFs, we generated dangling linker defects by disconnecting one tail of the linker group from the metal node and capping the resulting open metal node with capping agents. To ensure charge neutrality, a hydroxyl group was added as a capping agent in place of each removed linker if the linker is negatively charged. If the linker is charge-neutral, however, a water molecule was added as the capping agent in place of the removed linker. Hydroxyl groups and water molecules were used in a similar way as capping agents in structures with dangling linker defects. For example, in Figure 1a, the linker contains an O–C–O group attached to two metal sites via oxygen. In this case, a dangling linker will lead to the formation of O=C–O–, which will abstract H from surrounding water molecules. This could result in the formation of OH– ions that will attach to the metal site where the dangling linker formed and a free water molecule will attach to the other metal site. In this way, the overall system is still charge-neutral. A similar approach was applied to linker vacancies.

Figure 1 demonstrates some defective MOF structures containing linker vacancies. The atomic positions of these defective MOF structures were optimized using plane-wave DFT calculations with the VASP code and atomic charges were assigned by the DDEC6 method.^23^ For defect structure geometry optimization the cell shape and cell volume were kept fixed and the same functional and other computational details were used as those mentioned for the previous DFT calculations. To get comparative insights into the structural properties of defect-free-rigid structures and defective structures, their properties are compared in Tables S5 and S6.

### 2.3 MD-MC Hybrid Simulations of Water Uptake in MOFs

In our molecular simulations, we adopted the TIP4P^30^ model for water. This widely tested water model has a saturation vapor pressure of *P*_0_ = 4100 Pa at 298 K.^17,31^ Lennard-Jones (LJ) parameters for the framework atoms were taken from the Universal Force Field (UFF).^32^ van der Waals interactions between framework atoms and adsorbates were described by combining parameters from UFF for MOF atoms and from the TIP4P force field for water using the Lorentz–Berthelot mixing rules. As described in the previous section, atomic charges for the framework atoms were estimated using the DDEC6 method. All electrostatic interactions were calculated using the Ewald summation method.^33^

Molecular simulation of water adsorption using Grand Canonical Monte Carlo (GCMC) is often more challenging than similar simulations of nonpolar adsorbates.^17,34^ The flexibility of bound hydroxyl groups or water molecules can play a strong role in the adsorption of water.^35^ To address both of these issues, we performed molecular simulations using a Molecular Dynamics-Monte Carlo (MD-MC) hybrid method.^36^ The MD-MC hybrid scheme used in this study includes two steps: (1) In the MD step, an initial configuration of adsorbates with a number of adsorbate molecules approximately equivalent to the saturation loading is simulated in an NVT ensemble, allowing the movement of adsorbate and capping agents (hydroxyl group/water molecules) at defect sites, and (2) using the equilibrated adsorbate configuration from the MD step, Grand Canonical Monte Carlo (GCMC) is used to predict the equilibrium water uptake at specified pressure conditions with the MOF and capping agents assumed to be rigid.

**Figure 2:**
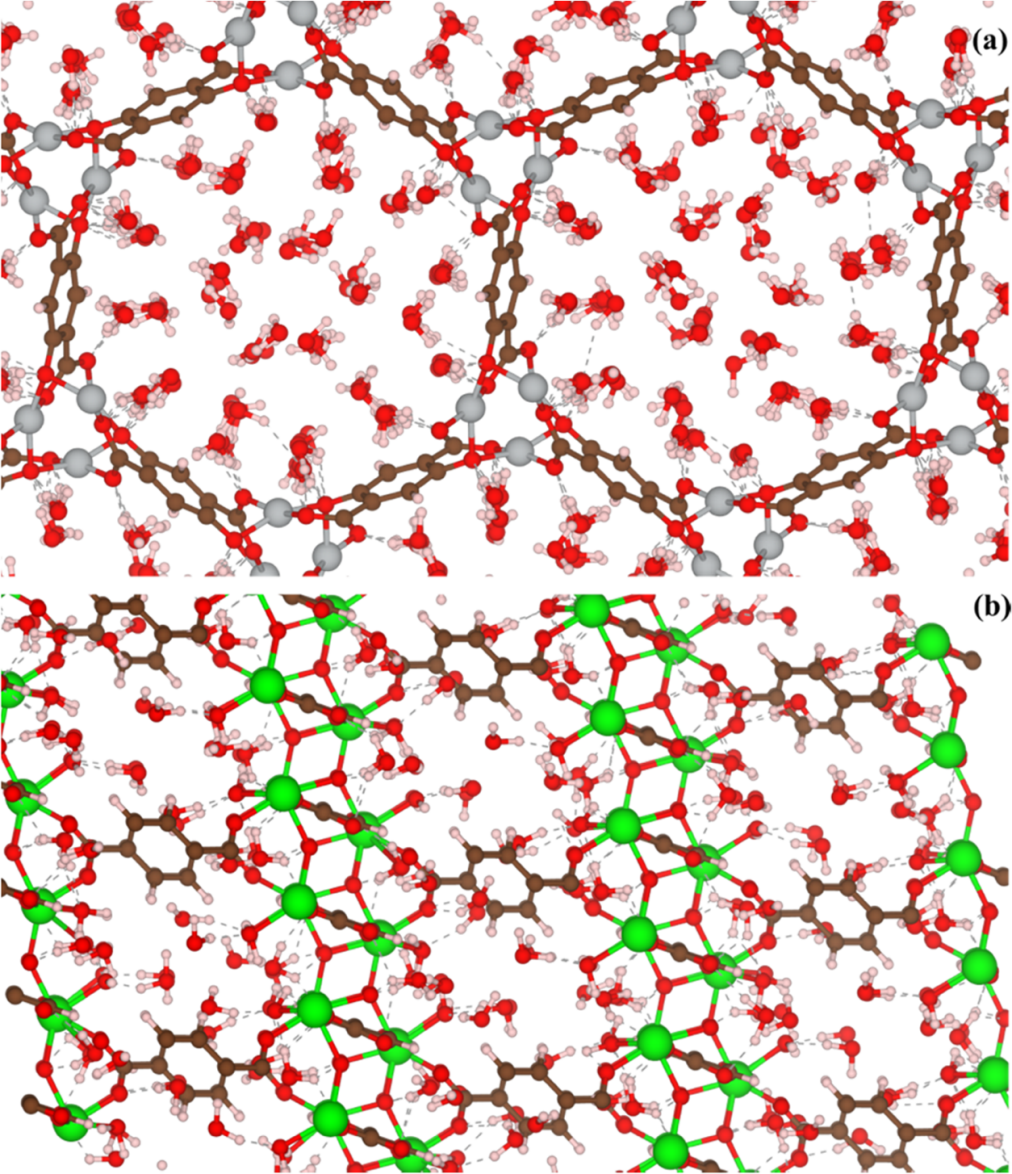
Snapshots of equilibrated adsorbate configurations from MD, including (a) water cluster formation in the pristine form of LECQEQ and (b) water cluster formation in ZONBAH containing linker vacancies through H-bonding with hydrophilic groups at defect sites.

In our MD-MC hybrid scheme, we used the LAMMPS_INTERFACE^37^ package to generate input files for LAMMPS^38^ for MD simulations. For pristine MOFs, MD involved only movement of adsorbate molecules, and all framework atoms were considered rigid. In defective MOFs, MD incorporated flexibility for defects with the movement of adsorbate molecules and capping agents (hydroxyl group/water molecule) at defect sites. The initial configuration of adsorbate molecules was energy-minimized using the *cg* style in LAMMPS with the mentioned degrees of freedom in the MOFs modeled using the UFF. MD simulation in the NVT ensemble was performed at 298 K with a time step of 1 fs and a production period of 1 ns in LAMMPS. Figure 2 shows an example of water cluster formation through H-bonding observed in these MD simulations. Each GCMC state point is based on a single MD snapshot. We have not explored the effects of averaging over multiple MD snapshot starting points because of the computational burden of these calculations.

Using equilibrated adsorbate configurations from the MD step, GCMC was performed using the RASPA package.^39^ For GCMC simulations of pristine and defective MOFs, all framework atoms were considered rigid, including hydroxyl groups or water molecules that are capping agents for defect sites. All Lennard-Jones potentials were truncated at a cutoff of 10 Å with analytical tail correction terms. We tested several LJ cutoff values (10, 11, and 12 Å) and did not observe any significant change in converged adsorption properties. Previous studies have also indicated that an LJ cutoff of 10 Å is adequate to get well-converged results.^40,41^ Random translation, rotation, reinsertion, and swap moves with equal probability along with identity change were attempted in the simulation cell. For all pressure points (*P*/*P*_0_ = 0.1, 0.2, 0.3, 0.5, 0.7, 0.8, 1), we ran GCMC simulations at 298 K with 1 million equilibration cycles and 10 million production cycles or for a simulation time of 400 h on our computer resources, whichever was less. For moderate to high water pressures, we found a number of simulations terminated because of our time requirement as simulations tend to run slower under these conditions. Figures S3 and S4 show extensive convergence data for our simulations, indicating that in almost all cases, clear evidence of convergence was observed. Test calculations showed that our MD-MC hybrid scheme accelerates convergence of this simulation compared to pure GCMC approach beginning from an empty structure, typically by a factor of 5–10.

## 3 Results and Discussion

### 3.1 Identifying Defective MOFs Using Water Uptake in Pristine MOFs

We first used the MD-MC hybrid scheme described in the previous section to estimate the water uptake in the pristine (i.e., defect-free) structures of the 35 MOFs listed in Table 2. In Section 1 we mentioned the experimental classification definitions for “low kinetic stability” and “unstable” MOFs. Of the 35 MOFs we selected, 12 are in the “low kinetic stability” category and 7 are in the “unstable” category. The remaining 16 MOFs came from the ML model predictions of Batra et al.,^20^ which does not distinguish between materials with “low kinetic stability” and materials that are “unstable” (see Table S1). In discussing the stability of MOFs with respect to water, it is important to distinguish between hydrophobic and hydrophilic MOFs. Hydrophobic MOFs typically do not have polar centers such as hydrogen bonding groups or uncoordinated metal sites, resulting in negligible water uptake.^31,42^ On the other hand, hydrophilic MOFs typically include polar groups and/or unsaturated metal sites that drive significant water uptake.^19^ Water adsorption–desorption isotherms provide a direct way to characterize the hydrophilicity/hydrophobicity of MOFs.^43^ We predicted the equilibrium water uptake in pristine MOFs at relative humidity (RH) ranging from 10 to 100%. We classified MOFs as being hydrophobic (hydrophilic) based on whether their water uptake was less (more) than 50% of the saturation water uptake at a threshold humidity. We used a threshold of 50% RH for “unstable” MOFs, 70% RH for “low kinetic stability” MOFs, and 50% RH for MOFs from the Batra et al.^20^ database. Later in this section, we explain our reasoning behind choosing these thresholds.

**Table 2:** List of Hydrophilic and Hydrophobic Pristine MOFs Identified from Simulated Water Adsorption, as Described in the Text

hydrophilic MOFs		hydrophobic MOFs	
BIBXUH	MUBWEO	ATOXAJ	VOXREI
FIJDOS	NAVJAW	BEYSEF	WONYEG
FIQCEN	QOWRAV	FOFCEL	WONYIK
GOTFED	TOPMIU_Co	GOPZIX	YUVSUE
IDIWIB	TOPMIU_Ni	GUBJEV	ZONBAH
IDIWOH	VOGTIV	OLEKUM	
LASYOU	VOXRAE	PARPII	
LECQEQ	WONYAC	POQNAN	
MEZNAJ	ZORFAQ	TOPMIU_Zn	
MIBQAR	QAVWAN_NO_CH3		
CIDKUX			

**Figure 3:**
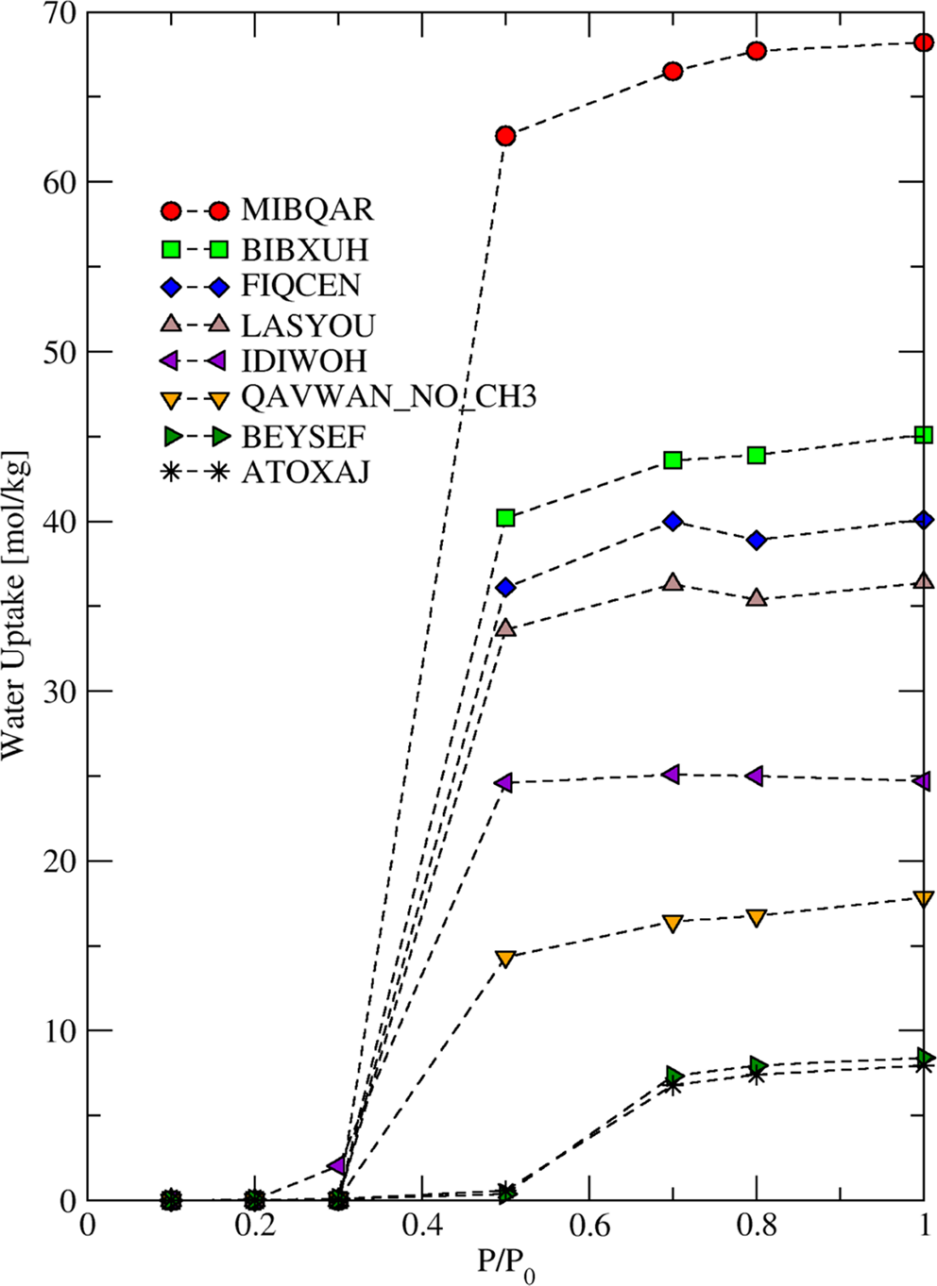
Water adsorption isotherms from GCMC simulations in representative pristine MOFs at 298 K.

Figure S1 shows a simulated water adsorption isotherm in all 35 pristine MOFs. Figure 3 shows the water adsorption isotherm in some representative pristine MOFs. Twenty-one of the 35 simulated materials are seen to be hydrophilic in their pristine form, for example, FIQCEN and IDIWOH, but others such as ATOXAJ are predicted to have negligible water uptake at 50% RH in their pristine form. Table 2 lists the MOFs identified as hydrophilic and hydrophobic via this analysis.

Of the 35 MOFs we simulated as pristine structures, 14 were identified as hydrophobic. We reiterate that all of the MOFs we simulated were previously classified by experimentally based methods or ML predictions as having low kinetic stability or being unstable with respect to water. This classification is inconsistent with a MOF being hydrophobic, so for the 14 hydrophobic MOFs we have identified the predictions of our molecular simulations are in conflict with experimental observations. We hypothesize that this discrepancy can be resolved by inferring the presence of defects in these MOFs that significantly alter the adsorption of water. We explore this hypothesis in detail below. Twenty-one of the pristine MOFs we simulated are predicted to be hydrophilic within our description. Because it is possible that the experimentally observed water-induced degradation occurs readily upon water adsorption in these hydrophilic materials, our results do not provide any insight into the presence or absence of defects in these materials.

MOFs from the “unstable” class mentioned above have been reported to be sensitive to “small amounts of moisture in the vapor phase”. We considered threshold values of 30 and 50% RH to describe this set of materials. Selecting a threshold of 30% RH would classify all 7 “unstable” pristine MOFs as hydrophobic, suggesting that defects in those MOFs would explain their observed instability at low to moderate humidity. If instead the threshold is chosen as 50% RH, then 3 of the 7 “unstable” pristine MOFs would be classified as hydrophilic, and our approach cannot yield any information about the potential presence or role of defects. Because the 50% RH threshold yields a more conservative interpretation, we chose this approach. The MOFs from the “low kinetic stability” class are known to be sensitive to exposure to water at “high humidity”. To reflect this qualitative description, we used a higher threshold of 70% RH for these materials. We have also considered 16 MOFs from the work of Batra et al.,^20^ which used an ML model that did not distinguish between the subclasses of stability described above. No experimental data are available to assess the water stability of any of these materials. Based on this lack of information, we used a threshold of 50% RH as a parsimonious choice. Although the humidity thresholds we chose are reasonable choices, we acknowledge that they are not quantitative and that variations in these choices could also be reasonable.

### 3.2 Influence of Linker Vacancy Defects on Water Adsorption in MOFs

The work of Chen et al. is one example where the presence of chemically plausible point defects in a MOF, DMOF-1, was shown to allow water cluster formation in a material that is hydrophobic in its pristine form.^9^ To test whether a similar mechanism could account for the observed lack of water stability for the 14 MOFs that we identified above as hydrophobic in their pristine form, we performed simulations examining the role of defects for each material. No information about the nature or concentration of defects in these materials is available experimentally, so we first generated chemically plausible models of point defects in each material. We then compared the water uptake in defective and flexible (incorporating the flexibility of bound hydroxyl groups or water molecules) MOF structures with predictions made for rigid defect-free structures.

**Figure 4:**
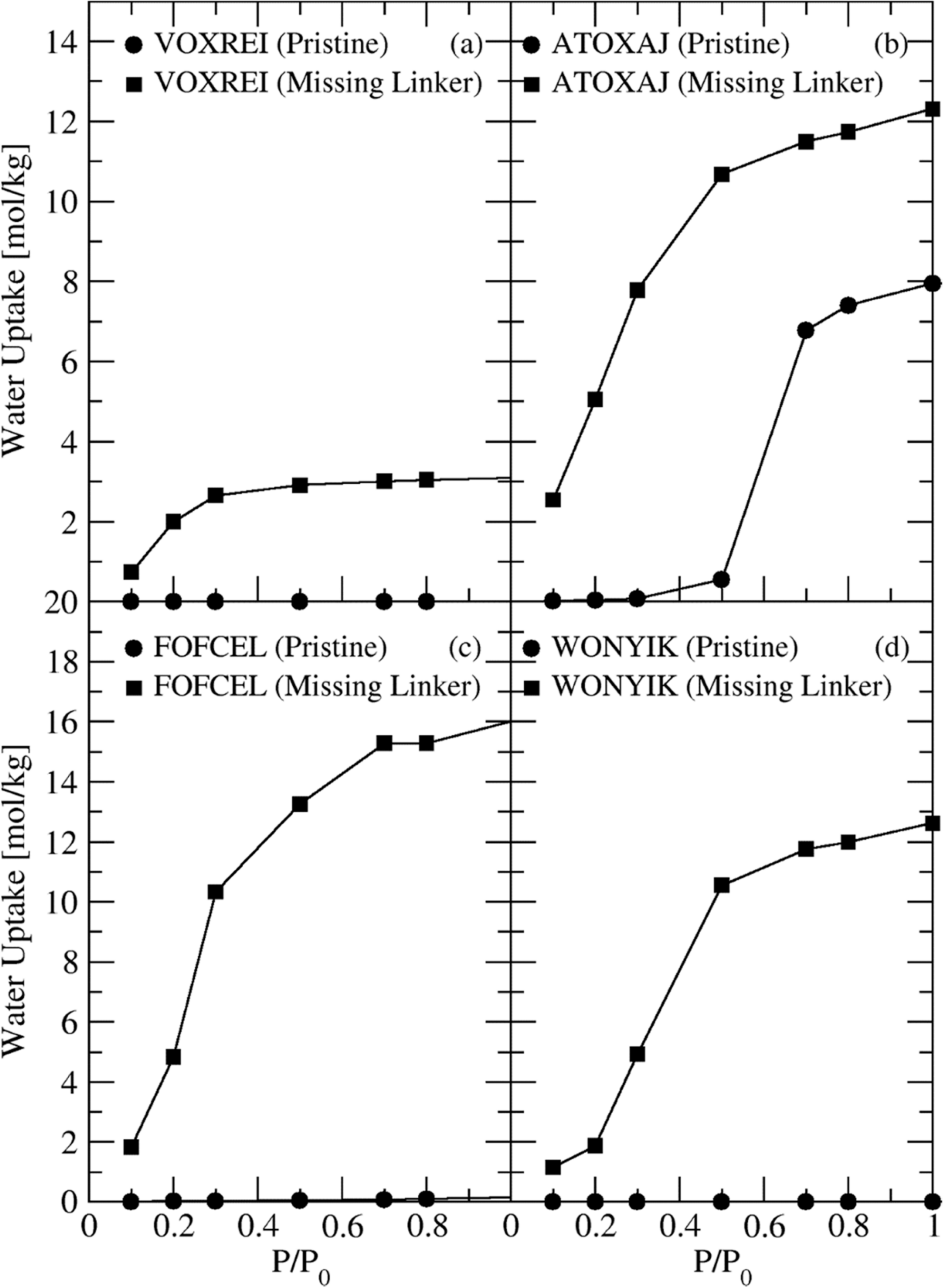
Simulated water adsorption isotherms at 298 K in several pristine MOFs and the corresponding MOF with one missing linker per unit cell: (a) VOXREI, (b) ATOXAJ, (c) FOFCEL, (d) WONYIK.

Figure 4 compares the water uptake in several pristine MOFs and the corresponding defective MOF with one missing linker from a 1 × 1 × 1 unit cell. Defective MOFs have a larger pore volume (see Table S6) and, more importantly, include the presence of hydrophilic groups. These hydrophilic groups promote H-bonding and thus water cluster formation. In all of the cases shown in Figure 4, the presence of defects leads to a significant increase in water uptake at low to moderate partial pressures relative to the pristine materials, imparting a hydrophilic nature to MOF. Eleven out of 14 MOFs identified as being hydrophobic in Table 2 become hydrophilic (using the definition given above) after introducing linker vacancy defects. This observation suggests that the presence of defects in these 11 MOFs accounts for the experimentally observed instability of the MOFs to water exposure. Two MOFs, GUBJEV and OLEKUM, did not show a significant increase in water uptake at 50% RH after introducing one linker vacancy per unit cell (see Figure S2). RH In GUBJEV, the hydrophobic pristine MOF has two types of linkers present. We only simulated linker vacancies associated with the smaller of these two linkers, which led to a moderate increase in water uptake compared with its pristine form. It is possible that the removal of the larger linker would create more hydrophilic sites at metal nodes and further increase water uptake, but we did not test this in our simulations. In OLEKUM, our simulations used one linker vacancy per unit cell, as for the other materials we simulated. Because of the large unit cell of the OLEKUM complex, this choice corresponds to a defect concentration of 0.04. It may be that at higher defect concentrations, more significant increases in water uptake occur, but we did not explore this directly because of the significant computational effort associated with simulating this MOF.

Introducing a linker vacancy defect in TOPMIU_Zn gave a structure that was not connected as a continuous framework, which we deemed unphysical. We therefore did not use this structure further. Water isotherms for 13 defective MOFs are shown in Figure S2. Given the scarce availability of data regarding the presence of defects in real MOFs, this collection of 11 materials that become hydrophilic due to the inclusion of point defects represents a considerable expansion of the set of MOFs for which the presence of defects can be inferred.

### 3.3 Influence of Defect Concentration on Water Adsorption in MOFs

The adsorption of water in MOFs can potentially be influenced by the number of defects that are present. To probe this effect, we performed simulations for some structures as a function of the density of the missing linkers. Detailed models of ZIFs have shown that in some cases clustering of defects is preferred during the formation of defects by some mechanisms.^28^ In the absence of information like this for the materials we simulated we varied the defect density by using simulations with a single defect in the simulation volume.

**Figure 5:**
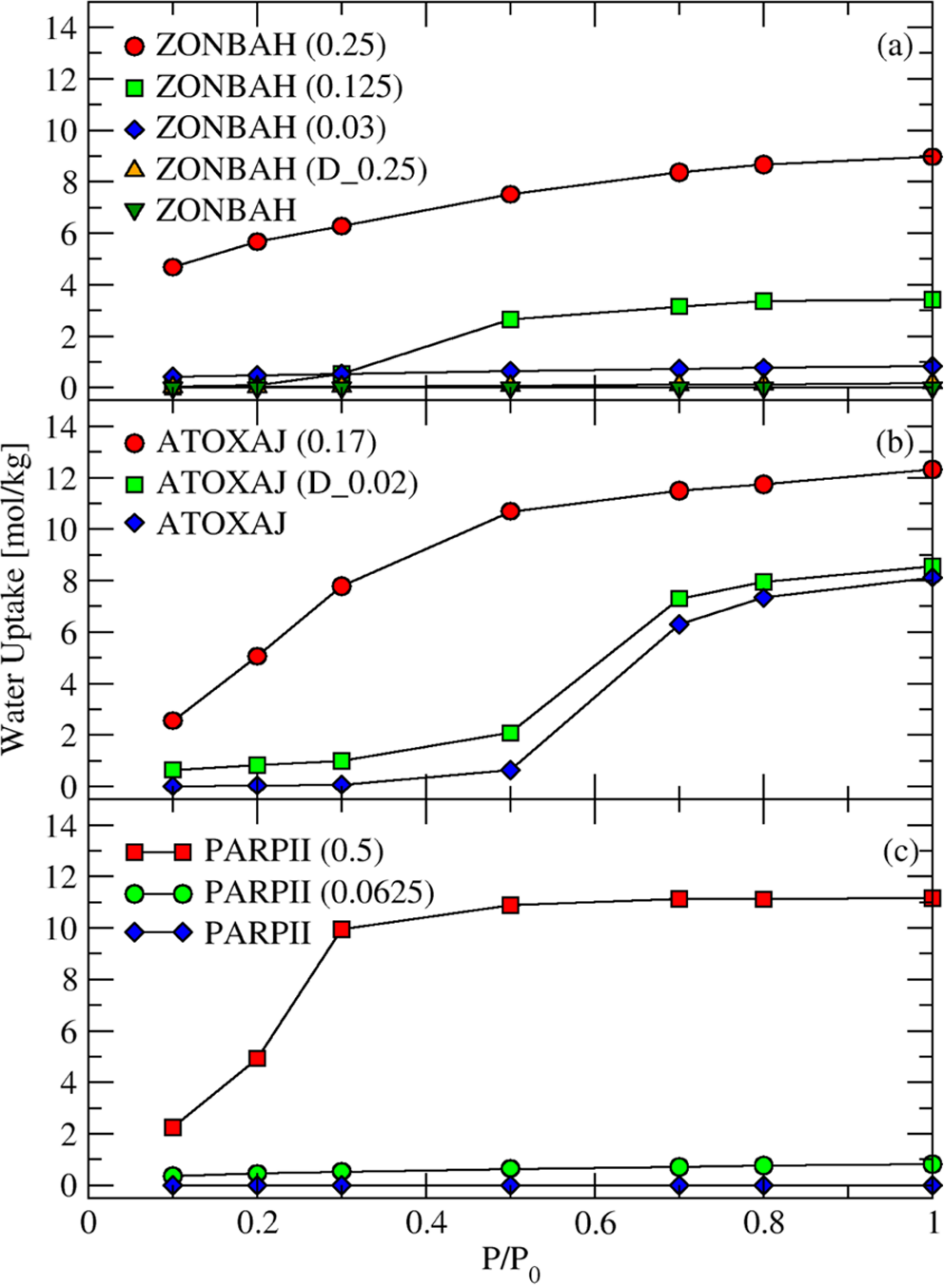
Simulated water adsorption isotherms at 298 K in MOFs with dangling linker and missing linker defects for (a) ZONBAH, (b) ATOXAJ, and (c) PARPII. ZONBAH (D_0.25) represents a dangling linker concentration of 0.25, and ZONBAH (0.25) represents a missing linker vacancy concentration of 0.25 for.

Figure 5 compares the water adsorption isotherms in pristine MOF structures and defective structures with varying defect concentrations. In Figure 5a, pristine ZONBAH is hydrophobic, as it shows no water uptake over the entire relative pressure range. A defect concentration of 0.03 in ZONBAH corresponds to one missing linker from a 2 × 2 × 2 supercell and a defect concentration of 0.125 indicates one missing linker from a 1 × 2 × 1 supercell. Both defect concentrations impart hydrophilicity to the MOF. The water loadings increase as the defect concentration is increased, with the highest water loadings when defect concentration is 0.25. Not surprisingly, the presence of more hydrophilic groups allowing strong interactions between adsorbed water and defect sites leads to increased water nucleation in pore. The increase in pore volume associated with defects increases the saturation capacity for water in the MOF. When ZONBAH was simulated with missing linker defects or dangling linker defects at the same concentration (0.25), the linker vacancy leads to a larger pore volume and more hydrophilic groups, leading to higher water uptake (see Table S6). In Figure 5b, the defect concentration of 0.17 in ATOXAJ corresponds to one missing linker among eight linkers in the unit cell and the defective MOF is hydrophilic. ATOXAJ (D_0.02), with a dangling linker defect concentration of 0.02, has a slightly higher accessible pore volume and surface area compared to pristine ATOXAJ and presence of hydrophilic groups at defect sites makes ATOXAJ (D_0.02) relatively more hydrophilic. Similarly in Figure 5c, pristine PARPII is hydrophobic, as it shows no water uptake over the entire relative pressure range. However, one missing linker from a 2 × 2 × 2 supercell of PARPII, i.e., PARPII (0.0625), introduces water molecules into the MOF, making it hydrophilic compared to its pristine form and subsequent nucleation of water molecules may lead to structural degradation.

## 4 Conclusions

Our aim in this paper has been to identify MOFs in which point defects are common in experimentally synthesized materials. Direct experimental characterization of the existence of defects in MOFs is challenging; therefore, it is valuable to expand the range of materials for which information on this topic is available. Our approach is based on the concept that in some situations, the adsorption properties of MOFs are changed in significant ways by the presence of defects. We focused on the adsorption of water since the stability of MOFs with respect to exposure to water has been established experimentally for many materials. We showed that in numerous materials that are known experimentally to be unstable with respect to water, molecular simulations based on pristine (i.e., defect-free) crystal structures predict the materials to be hydrophobic. This prediction is in conflict with experimental observations since hydrophobic materials should be resistant to water exposure. We further showed for numerous materials that the introduction of chemically plausible missing linker defects led to simulated water isotherms predicting hydrophilic behavior stemming from the nucleation of water clusters at defect sites. In total, we identified 11 materials with this behavior. We conclude that in the experiments that have been reported for these 11 materials, it is likely that defects were present that drove water adsorption and ultimately led to degradation of the materials. This work significantly expands the number of MOFs for which the presence of defects can be inferred from the experimental data. We hope that this outcome will lead to new directions for understanding the presence and properties of defects in a range of real materials.

It is important to clarify some conclusions that cannot be drawn from our results. We did not attempt to describe the mechanisms leading to instability with respect to water in the MOFs we studied as our focus was on understanding the presence (or absence) of defects. Some of the materials that we simulated were found to be hydrophilic in their pristine state. In this situation, our simulations cannot infer anything about the presence or absence of defects from experimental observations of instability with respect to water. The classification of the MOFs we studied as being unstable in water was based on extant experimental data.^18^ In the majority of MOFs, the number of reported measurements is small.^44^ Our predictions do not preclude the possibility that improved synthesis, handling, or activation methods could reduce the impact of defects on adsorption, rendering a MOF that was previously classified as unstable to water exposure as stable. Indeed, by giving a structural hypothesis for the source of water nucleation, our results may suggest experimental approaches to tackle this interesting challenge.

We noted in the Section 1 that the external surfaces of MOF crystals can potentially have different hydrophilicity than the interior pores of the MOF. For a pristine MOF that is hydrophobic (according to simulations) but unstable to water exposure experimentally, this observation raises the possibility that external surfaces rather than interior defects might drive degradation by water. We have not attempted to simulate the external surfaces of MOFs, in part because the atomic-scale structure of these surfaces is highly uncertain. This situation means that we cannot unambiguously rule out the impact of external surfaces on the water instability of nominally hydrophobic MOFs. Our simulations have demonstrated a chemically plausible route to water nucleation inside the pores of these MOFs due to point defects. It would be interesting to attempt experiments that could distinguish between these two factors. One possibility may be to perform experiments on batches made up of crystals of different sizes since the effects of external surfaces should be dictated by the surface-to-volume ratio, unlike the situation for defects in the bulk. An independent experimental strategy would be to adapt methods from MOF defect engineering to deliberately create high levels of internal defects. Both of these approaches would require methods that can assess the rate of degradation by water exposure, not simply a binary classification of whether degradation occurred.

In this study, we hypothesized that the presence of defects in MOFs can induce water nucleation that leads to water uptake, imparting hydrophilicity to MOF that contributes to MOF degradation. Our simulation scheme incorporates the flexibility of capping agents (OH^–^ and H_2_O groups) at metal sites, but not the flexibility of other degrees of freedom in MOFs. This approach assumes that this local flexibility is critical to the formation of H-bonding at defect sites (Figure 2) but that other aspects of MOF flexibility are less critical to the initial nucleation of water clusters. Adsorbate-induced flexibility can affect a range of MOF properties, so detailed models that aimed to explore the mechanisms of water-induced degradation would likely need to account for the flexibility of the entire framework.

Our results have interesting implications for the use of high-throughput calculations for predicting molecular adsorption in MOFs. A variety of studies using molecular simulations of adsorption isotherms or ML models trained from underlying molecular simulation data have been reported,^45−50^ but to date, all of these efforts have used simulations of pristine MOFs. The simulations we have reported in this paper significantly expand the number of MOFs that have been treated with molecular simulations in which the presence of defects makes significant differences in the adsorption isotherms of water. We emphasize that these effects will occur not only for water adsorption but also for the adsorption of any molecules expected to strongly interact with defects or for the adsorption of mixtures containing even small amounts of these molecules.

## Supporting Information Available

Additional data and computational results (PDF)
Example input files for DFT, MD, and GCMC simulations, CIF files for the 35 pristine MOFs and 13 defective MOFs; complete collection of data from screening of pristine MOFs and corresponding defective MOFs in a simplified form; the XLSX file contain structural properties and water isotherm data at 298 K for all studied MOFs obtained from detailed GCMC simulations; and list of 89 low kinetic stability or unstable MOFs (ZIP)

## Notes

Notice of Copyright: This work has been authored by UT-Battelle, LLC, under contract DE-AC05–00OR22725 with the US Department of Energy (DOE). The publisher acknowledges the US government license to provide public access under the DOE Public Access Plan (http://energy.gov/downloads/doe-public-access-plan).
