# Supplementary material for: Probing Structural Defects in MOFs Using Water Stability
Source: J Phys Chem C Nanomater Interfaces. 2024 Feb 23;128(9):3975–84. doi: 10.1021/acs.jpcc.3c07497 (PMC10926153; doi:10.1021/acs.jpcc.3c07497)
Supplement: Supplementary file 1 — jp3c07497_si_001.pdf [file jp3c07497_si_001.pdf]

Supporting Information for
Probing Structural Defects in MOFs Using Water Stability

Shubham Jamdade[1], Zhenzi Yu[1], Salah Eddine Boulfelfel[1], Xuqing Cai[1], Raghuram Thyagarajan[1], Hanjun Fang[1] and David S. Sholl[1,2*]

[1]School of Chemical & Biomolecular Engineering, Georgia Institute of Technology, Atlanta, Georgia 30332-0100, United States
[2]Oak Ridge National Laboratory, Oak Ridge, TN 37830, United States

*Corresponding author email: shollds@ornl.gov

I. **MOFs and Their Structural Properties**
II. **Identifying hydrophilic-hydrophobic MOFs**
III. **Water adsorption isotherms in pristine and corresponding defective MOFs**
IV. **Convergence plots for GCMC simulations for pristine MOFs**
V. **Convergence plots for GCMC simulations for defective MOFs**

I. MOFs and Their Structural Properties

The data used to make the figures in this paper are all included in the ZIP file also available in the SI. For all pristine and corresponding defective MOFs, water adsorption isotherms are listed in Adsorption_Water.xlsx and structural properties are in Structural_Properties.xlsx. A list of 89 low kinetic stability or unstable MOFs obtained from dataset by Burtch et al.[1] and Batra et al.[2] along with DOI reference for each of the MOF is collected in the Low_water_stable_MOFs.xlsx. MOF CIF files for pristine and corresponding defective structures are in the ZIP file in SI. Example input for DFT, MD and GCMC simulations can also be found in the ZIP file.

Table S1: List of 35 computation ready MOFs and their classification category

MOF category		
Unstable	Low Kinetic Stability	Batra et al.[2] database
ATOXAJ	BIBXUH	FOFCEL
BEYSEF	CIDKUX	GOPZIX
IDIWOH	FIJDOS	GOTFED
LASYOU	FIQCEN	GUBJEV
MIBQAR	IDIWIB	MEZNAJ
OLEKUM	LECQEQ	MUBWEO
YUVSUE	NAVJAW	POQNAN
	PARPII	TOPMIU_Co
	QAVWAN_NO_CH3	TOPMIU_Ni
	QOWRAV	TOPMIU_Zn
	VOGTIV	VOXRAE
	ZONBAH	VOXREI
		WONYAC
		WONYEG
		WONYIK
		ZORFAQ

In the following tables S2 and S3, Prefix UO before a property title represents structural property for corresponding MOF before full cell geometry optimization (i.e., for Unoptimized pristine MOF). Other property titles represent structural property for the MOF after full cell geometry optimization. LCD is the largest cavity diameter (Å), PLD is the pore limiting diameter (Å), SA is the gravimetric surface area (m^2/g), PV is accessible pore volume (cm^3/g), VF is the void fraction,

Table S2: List of 35 pristine MOFs with their LCD and PLD data before and after full cell geometry optimization using DFT.

MOFs	UO LCD (Å)	LCD (Å)	UO PLD (Å)	PLD (Å)
ATOXAJ	4.6	4.6	3.6	3.6
BEYSEF	5.6	4.8	4.7	4.1

BIBXUH	14.7	14.9	5.1	5.2
CIDKUX	9.2	7.5	8.3	6.6
FIJDOS	11.7	11.8	10.9	11.0
FIQCEN	13.2	13.4	6.7	6.7
FOFCEL	4.4	3.7	3.0	3.1
GOPZIX	3.6	2.7	1.5	1.5
GOTFED	5.1	-	2.8	-
GUBJEV	4.9	4.7	4.4	4.2
IDIWIB	7.5	7.5	6.3	6.3
IDIWOH	7.7	7.6	7.2	7.1
LASYOU	10.1	10.1	4.9	4.9
LECQEQ	11.5	11.3	10.7	10.5
MEZNAJ	4.1	3.1	2.1	2.2
MIBQAR	15.1	15.2	7.9	8.0
MUBWEO	4.5	4.5	1.4	1.3
NAVJAW	11.4	11.8	11.0	11.0
OLEKUM	6.9	6.9	5.7	5.7
PARPII	2.9	2.3	2.6	2.1
POQNAN	4.9	4.2	2.0	1.4
QAVWAN_No_CH3	6.4	6.4	4.8	4.8
QOWRAV	16.4	16.4	5.2	5.2
TOPMIU_Co	2.1	2.2	0.9	0.9
TOPMIU_Ni	2.1	2.8	0.9	1.0
TOPMIU_Zn	2.1	2.3	0.9	1.0
VOGTIV	11.6	11.8	10.8	11.0
VOXRAE	4.5	4.2	2.2	2.0
VOXREI	2.7	2.4	1.3	1.0
WONYAC	5.7	-	5.1	-
WONYEG	5.3	4.9	4.1	3.8
WONYIK	2.3	2.2	1.1	1.0
YUVSUE	5.8	4.8	4.6	4.0
ZONBAH	3.8	3.8	3.1	3.1
ZORFAQ	5.7	5.9	3.6	3.5

Table S3: List of 35 pristine MOFs with their surface area and pore volume data before and after full cell geometry optimization using DFT.

**MOF Code**	**UO SA (m^2/g)**	**SA (m^2/g)**	**UO PV (cm^3/g)**	**PV (cm^3/g)**	**UO VF**	**VF**
ATOXAJ	927	927	0.24	0.24	0.31	0.31
BEYSEF	1362	1059	0.31	0.22	0.36	0.30
BIBXUH	2853	2856	0.96	0.96	0.71	0.70
CIDKUX	1787	1340	0.59	0.37	0.62	0.49
FIJDOS	1383	1423	0.62	0.63	0.76	0.75
FIQCEN	2546	2566	0.86	0.86	0.76	0.75
FOFCEL	963	582	0.20	0.10	0.35	0.17

GOPZIX	153	0	0.04	0.00	0.06	0.00
GOTFED	413	324	0.11	0.09	0.15	0.12
GUBJEV	917	880	0.25	0.23	0.32	0.29
IDIWIB	1678	1712	0.48	0.51	0.51	0.54
IDIWOH	1917	1944	0.63	0.62	0.63	0.61
LASYOU	2626	2666	0.81	0.82	0.75	0.74
LECQEQ	1410	1249	0.61	0.54	0.73	0.70
MEZNAJ	219	59	0.07	0.02	0.10	0.03
MIBQAR	3709	3776	1.38	1.38	0.81	0.80
MUBWEO	189	219	0.07	0.08	0.10	0.11
NAVJAW	1432	1456	0.63	0.63	0.73	0.74
OLEKUM	2015	2041	0.56	0.56	0.57	0.58
PARPII	36	0	0.04	0.00	0.06	0.00
POQNAN	408	295	0.12	0.10	0.16	0.13
QAVWAN_No_CH3	1702	1702	0.43	0.43	0.48	0.48
QOWRAV	3117	3117	1.00	0.97	0.72	0.70
TOPMIU_Co	0	0	0.00	0.00	0.00	0.00
TOPMIU_Ni	0	2	0.00	0.01	0.00	0.01
TOPMIU_Zn	0	0	0.00	0.00	0.00	0.00
VOGTIV	1830	1887	0.80	0.84	0.73	0.75
VOXRAE	484	349	0.13	0.09	0.17	0.12
VOXREI	0	0	0.00	0.00	0.00	0.00
WONYAC	914	844	0.33	0.24	0.33	0.33
WONYEG	1178	1055	0.30	0.24	0.35	0.30
WONYIK	0	0	0.00	0.00	0.00	0.00
YUVSUE	1329	962	0.30	0.20	0.37	0.28
ZONBAH	425	473	0.10	0.11	0.17	0.19
ZORFAQ	1204	1126	0.32	0.29	0.37	0.34

Table S4:List of 13 defective MOFs with corresponding defect concentration in terms of number of linker vacancies and dangling linkers based on calculations with a 1×1×1 unit cell, 1×2×1 supercell and 2×2×2 supercell

**MOF code**	**No. of linkers in unit cell**	**Defect Concentration One missing/dangling linker per**		
		**1×1×1 unit cell**	**1×2×1 supercell**	**2×2×2 supercell**
ATOXAJ	6	0.17	0.08	0.02
BEYSEF	8	0.13	0.06	0.02
FOFCEL	4	0.25	0.13	0.03
GOPZIX	2	0.50	0.25	0.06
GUBJEV	6	0.17	0.08	0.02
OLEKUM	24	0.041	0.02	0.005
PARPII	2	0.50	0.25	0.06
POQNAN	4	0.25	0.13	0.03

VOXREI	6	0.17	0.08	0.02
WONYEG	4	0.25	0.13	0.03
WONYIK	3	0.33	0.17	0.04
YUVSUE	8	0.13	0.06	0.02
ZONBAH	4	0.25	0.13	0.03

In the following table S5 and S6 , a prefix 'P' before a property title represents a structural property for the corresponding pristine MOF and the prefix "D" before a property title represents a structural property for the defective MOF at the defect concentration specified in parentheses in the MOF name. LCD is the largest cavity diameter (Å), PLD is the pore limiting diameter (Å), SA is the gravimetric surface area (m^2/g), PV is accessible pore volume (cm^3/g), VF is the void fraction,

Table S5: LCD and PLD data comparison for pristine and corresponding defective structures

MOF Code	D_LCD (Å)	P_LCD (Å)	D_PLD (Å)	P_PLD (Å)
ATOXAJ (0.17)	5.5	4.6	3.5	3.6
ATOXAJ (D_0.02)	4.6	4.6	3.6	3.6
BEYSEF (0.13)	6.6	4.8	4.0	4.1
FOFCEL (0.25)	6.1	3.7	4.3	3.1
GOPZIX (0.5)	3.7	2.7	2.5	1.5
GUBJEV (0.17)	5.3	4.7	4.0	4.2
OLEKUM (0.08)	8.9	6.9	5.7	5.7
PARPII (0.5)	3.8	2.3	3.1	2.1
PARPII (0.06)	2.3	2.3	1.7	2.1
POQNAN (0.25)	5.1	4.2	2.5	1.4
VOXREI (0.17)	5.3	2.4	1.5	1.0
WONYEG (0.25)	6.6	4.9	4.4	3.8
WONYIK (0.33)	5.0	2.2	3.7	1.0
YUVSUE (0.13)	5.9	4.8	3.9	4.0
ZONBAH (0.25)	5.4	3.8	4.2	3.1
ZONBAH (0.13)	3.8	3.8	3.1	3.1
ZONBAH (0.03)	3.8	3.8	3.1	3.1
ZONBAH (D_0.25)	3.9	3.8	2.8	3.1

Table S6: Surface area and pore volume data comparison for pristine and corresponding defective structures

MOF Code	D_SA (m^2/g)	P_SA (m^2/g)	D_ PV (cm^3/g)	P_PV (cm^3/g)	D_VF	P_VF
ATOXAJ (0.17)	1103	927	0.28	0.24	0.32	0.31
ATOXAJ (D_0.02)	957	927	0.25	0.24	0.32	0.31
BEYSEF (0.13)	1152	1059	0.24	0.22	0.31	0.30
FOFCEL (0.25)	1664	582	0.37	0.10	0.52	0.17
GOPZIX (0.5)	264	0	0.07	0.00	0.09	0.00
GUBJEV (0.17)	1124	880	0.28	0.23	0.32	0.29

OLEKUM (0.08)	2238	2041	0.62	0.56	0.60	0.58
PARPII (0.5)	947	0	0.18	0.00	0.21	0.00
PARPII (0.06)	75	0	0.02	0.00	0.03	0.00
POQNAN (0.25)	861	295	0.21	0.10	0.24	0.13
VOXREI (0.17)	212	0	0.07	0.00	0.10	0.00
WONYEG (0.25)	2285	1055	0.55	0.24	0.51	0.30
WONYIK (0.33)	1259	0	0.34	0.00	0.39	0.00
YUVSUE (0.13)	1089	962	0.23	0.20	0.30	0.28
ZONBAH (0.25)	947	473	0.22	0.11	0.34	0.19
ZONBAH (0.13)	688	473	0.16	0.11	0.26	0.19
ZONBAH (0.03)	528	473	0.12	0.11	0.21	0.19
ZONBAH (D_0.25)	373	473	0.09	0.11	0.17	0.19

II. Identifying hydrophilic-hydrophobic MOFs

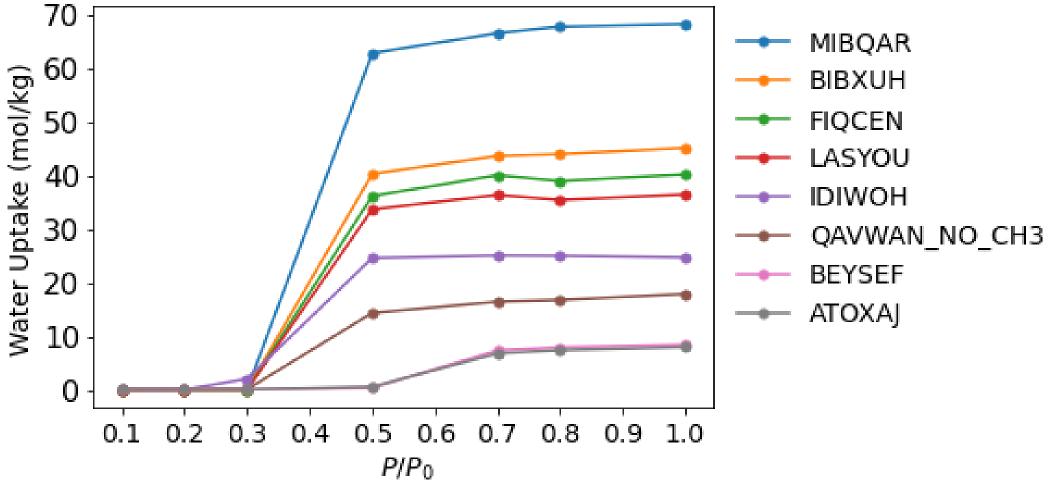

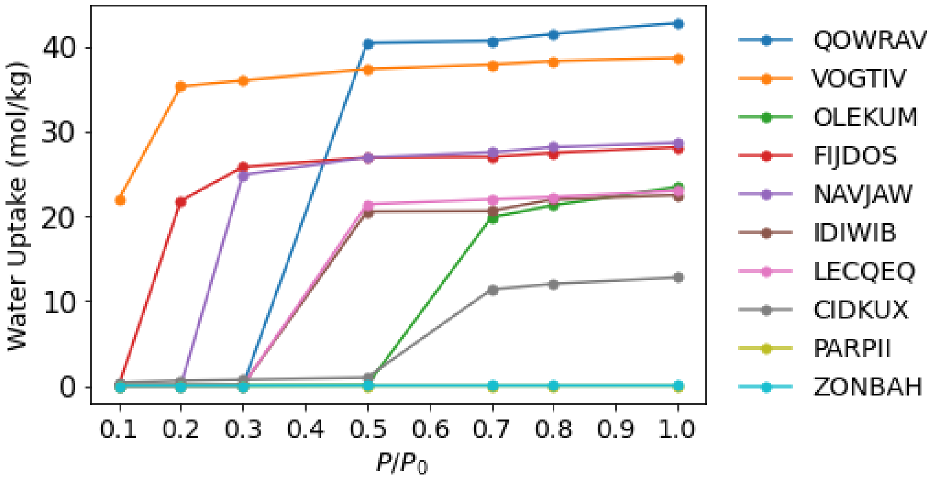

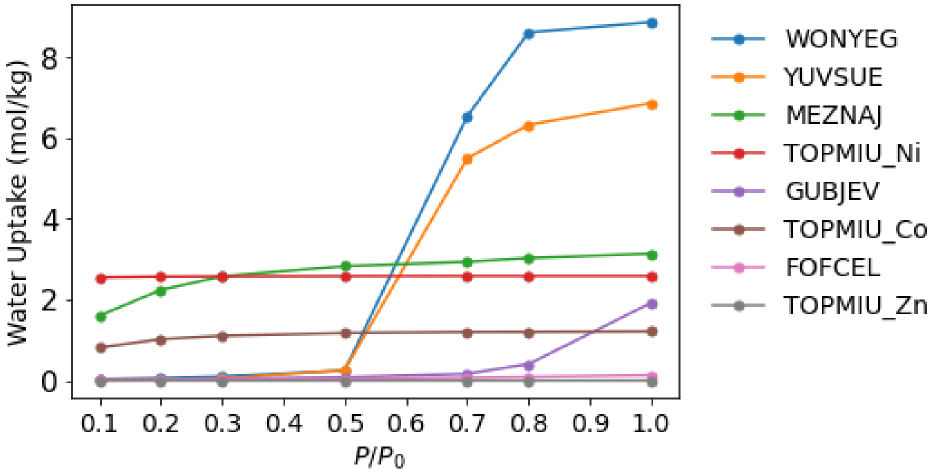

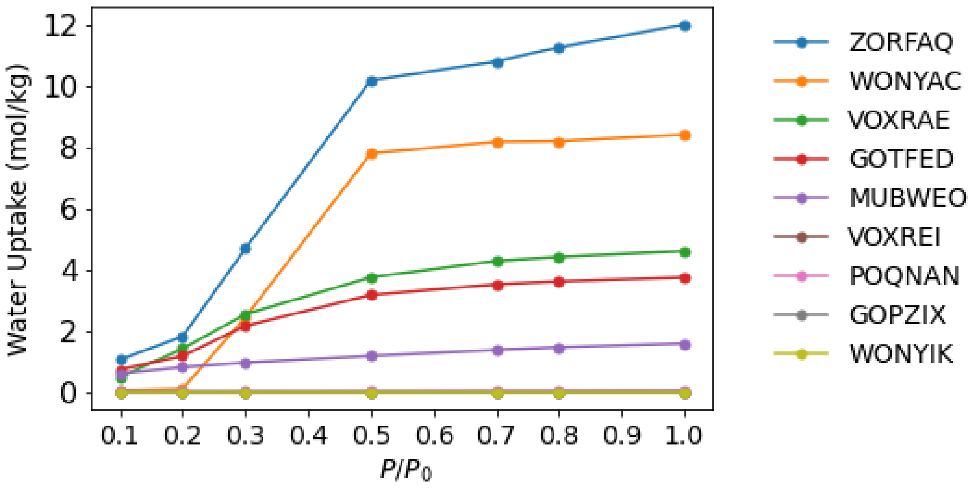

Figure S1: Water adsorption isotherms from GCMC simulations in 35 pristine MOFs at 298 K

III. Water adsorption isotherms in Pristine and corresponding defective MOFs

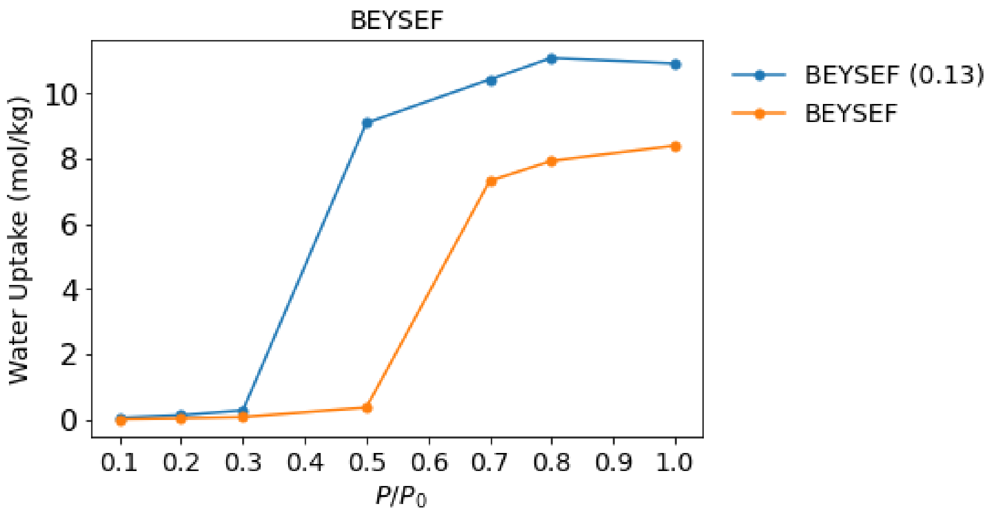

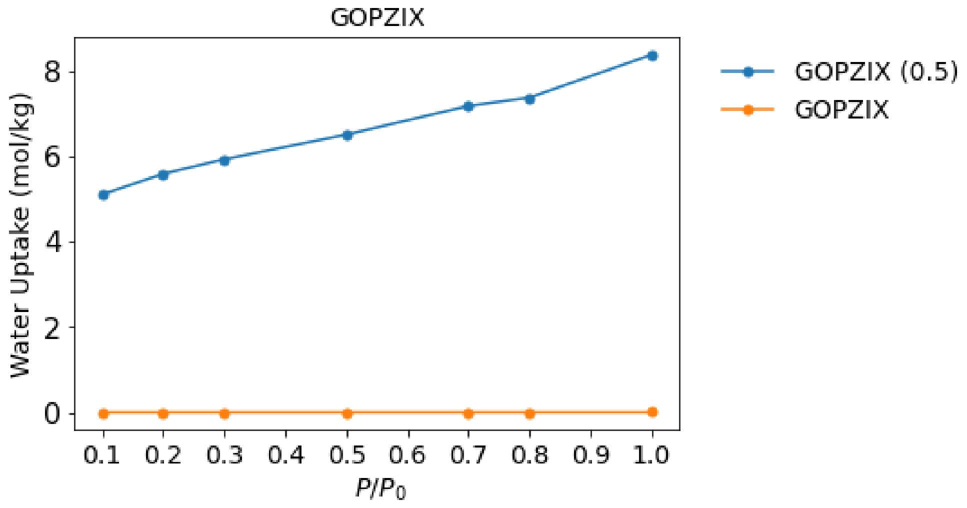

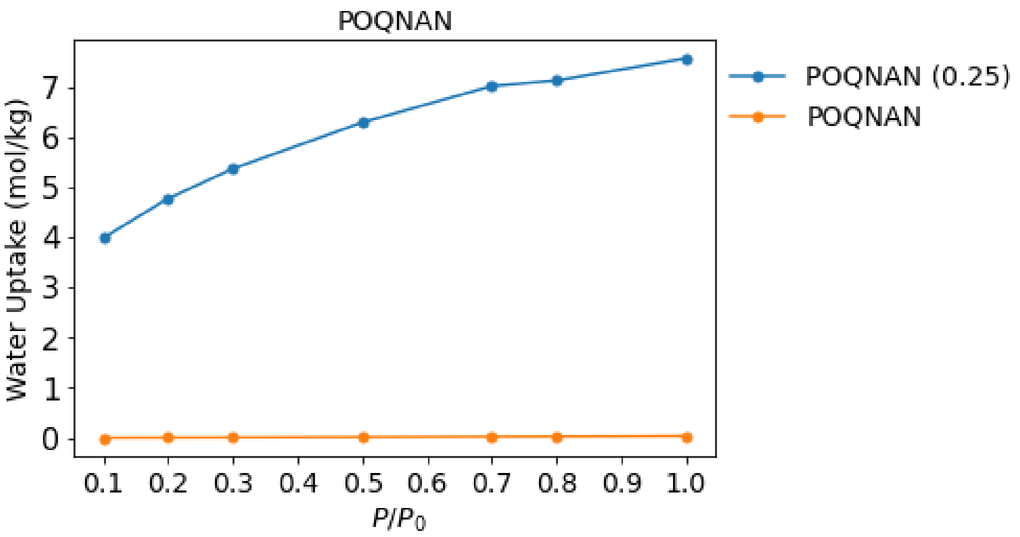
POQNAN
Water Uptake (mol/kg)
0
1
2
3
4
5
6
7
0.1
0.2
0.3
0.4
0.5
0.6
0.7
0.8
0.9
1.0
P/P_0
POQNAN (0.25)
POQNAN

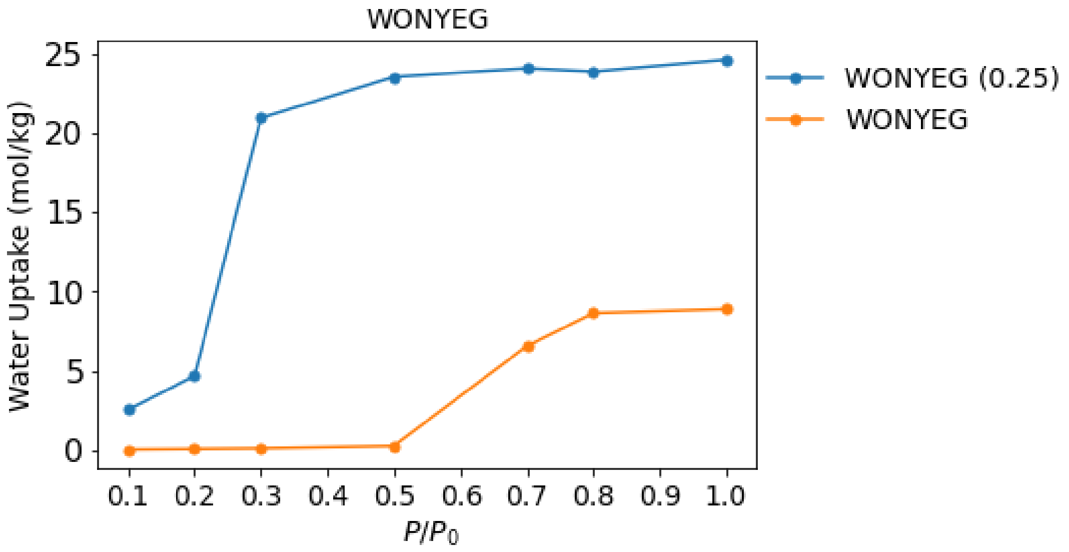
WONYEG
Water Uptake (mol/kg)
0
5
10
15
20
25
0.1
0.2
0.3
0.4
0.5
0.6
0.7
0.8
0.9
1.0
P/P_0
WONYEG (0.25)
WONYEG

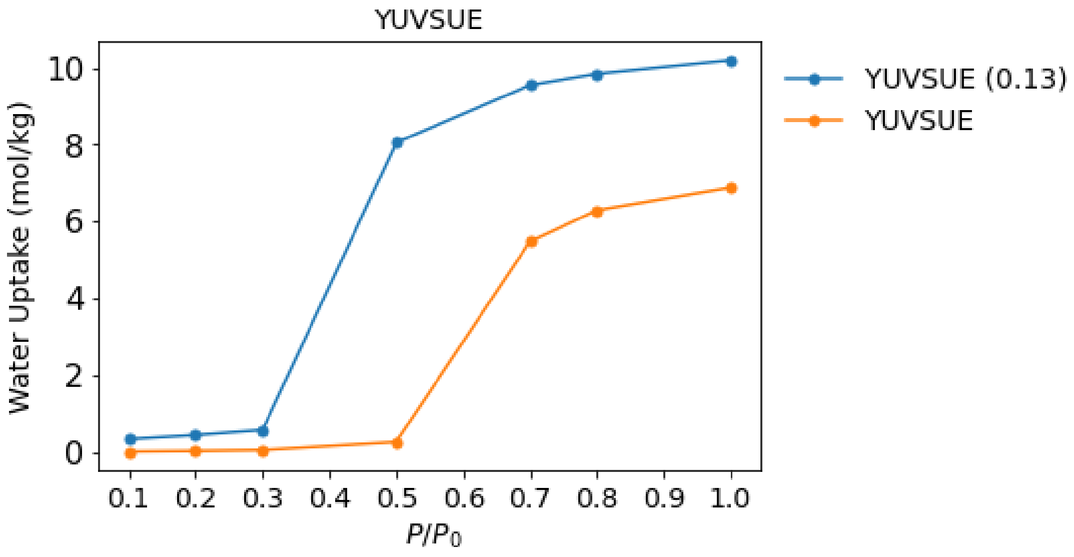
YUVSUE
Water Uptake (mol/kg)
0
2
4
6
8
10
0.1
0.2
0.3
0.4
0.5
0.6
0.7
0.8
0.9
1.0
P/P_0
YUVSUE (0.13)
YUVSUE

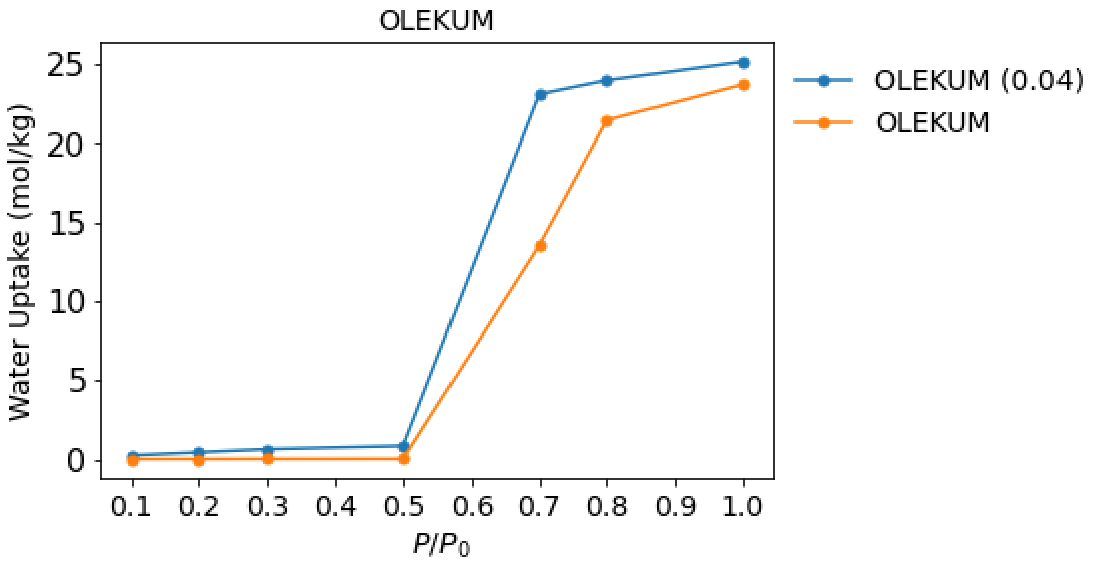
OLEKUM
Water Uptake (mol/kg)
0
5
10
15
20
25
0.1
0.2
0.3
0.4
0.5
0.6
0.7
0.8
0.9
1.0
P/P_0
OLEKUM (0.04)
OLEKUM

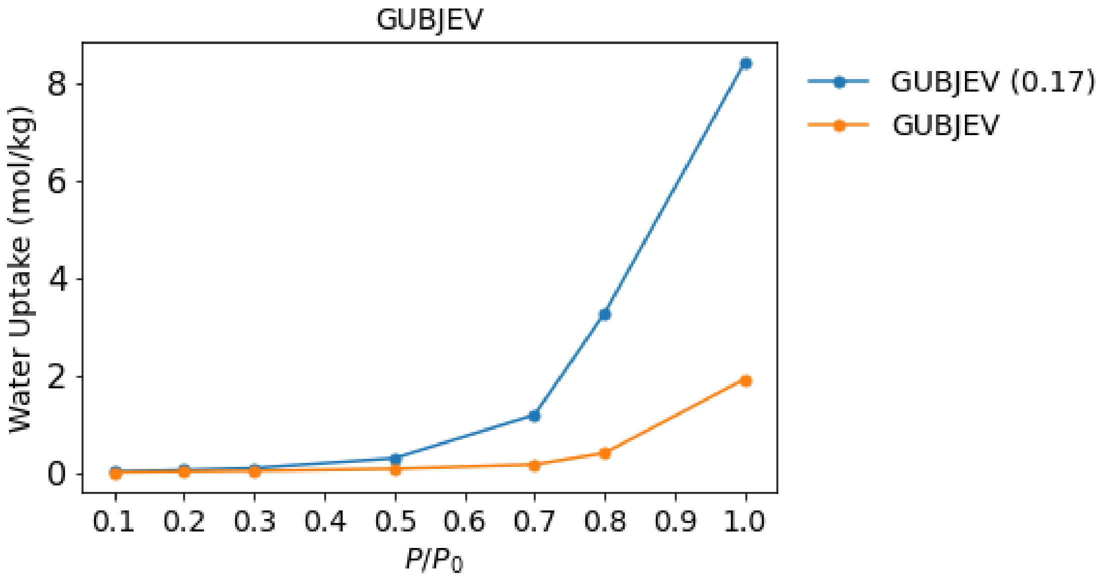
GUBJEV
Water Uptake (mol/kg)
0
2
4
6
8
0.1
0.2
0.3
0.4
0.5
0.6
0.7
0.8
0.9
1.0
P/P_0
GUBJEV (0.17)
GUBJEV

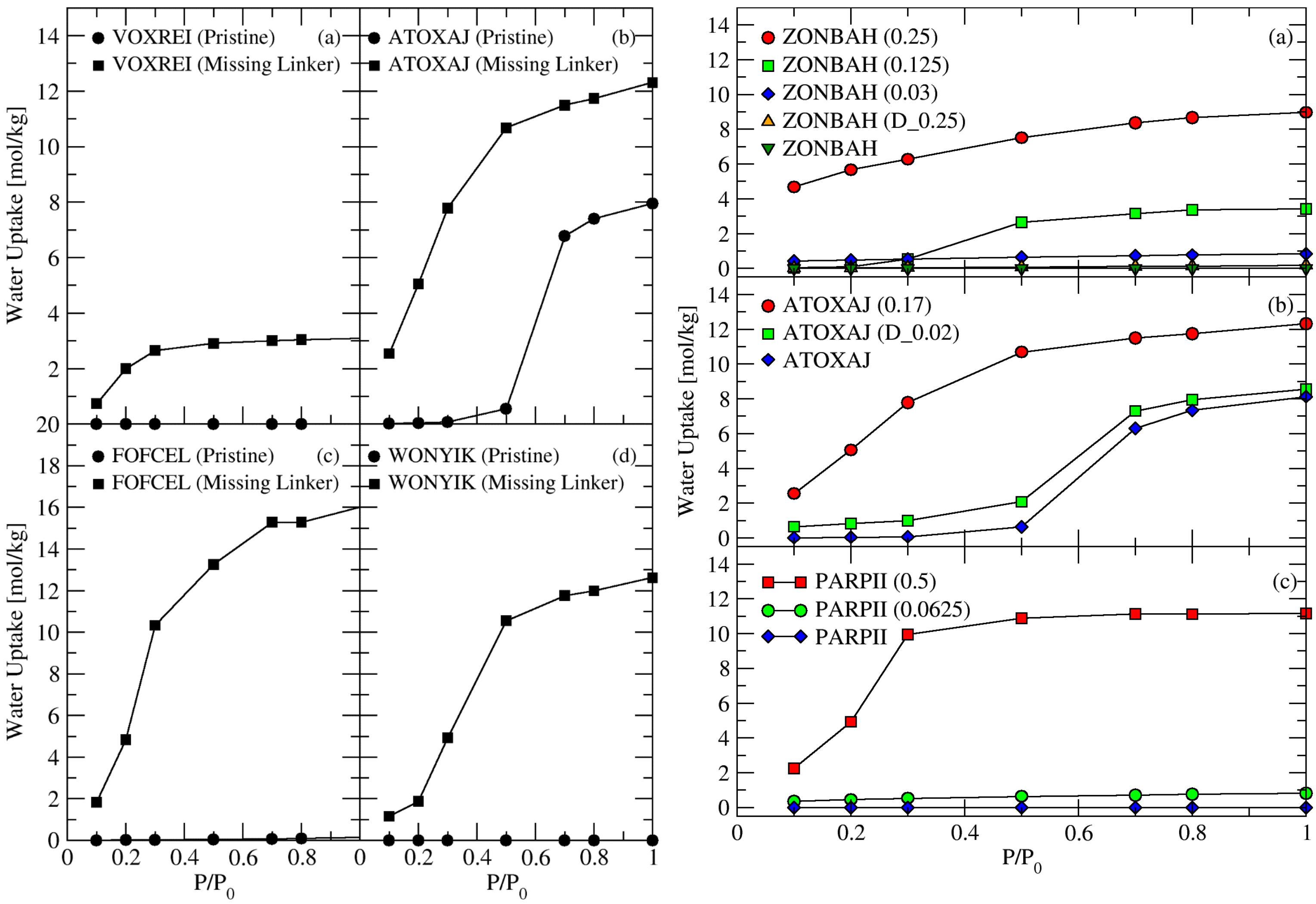

Figure S2: Water adsorption isotherms from GCMC simulations in pristine and corresponding defective MOFs with one missing linker from a unit cell at 298 K. "MOF Code (defect concentration)" represents concentration of missing linkers.

IV. Convergence plots for GCMC simulations for pristine MOFs

Convergence plots for all pristine MOFs at pressure point $P/P_0 = 0.1$

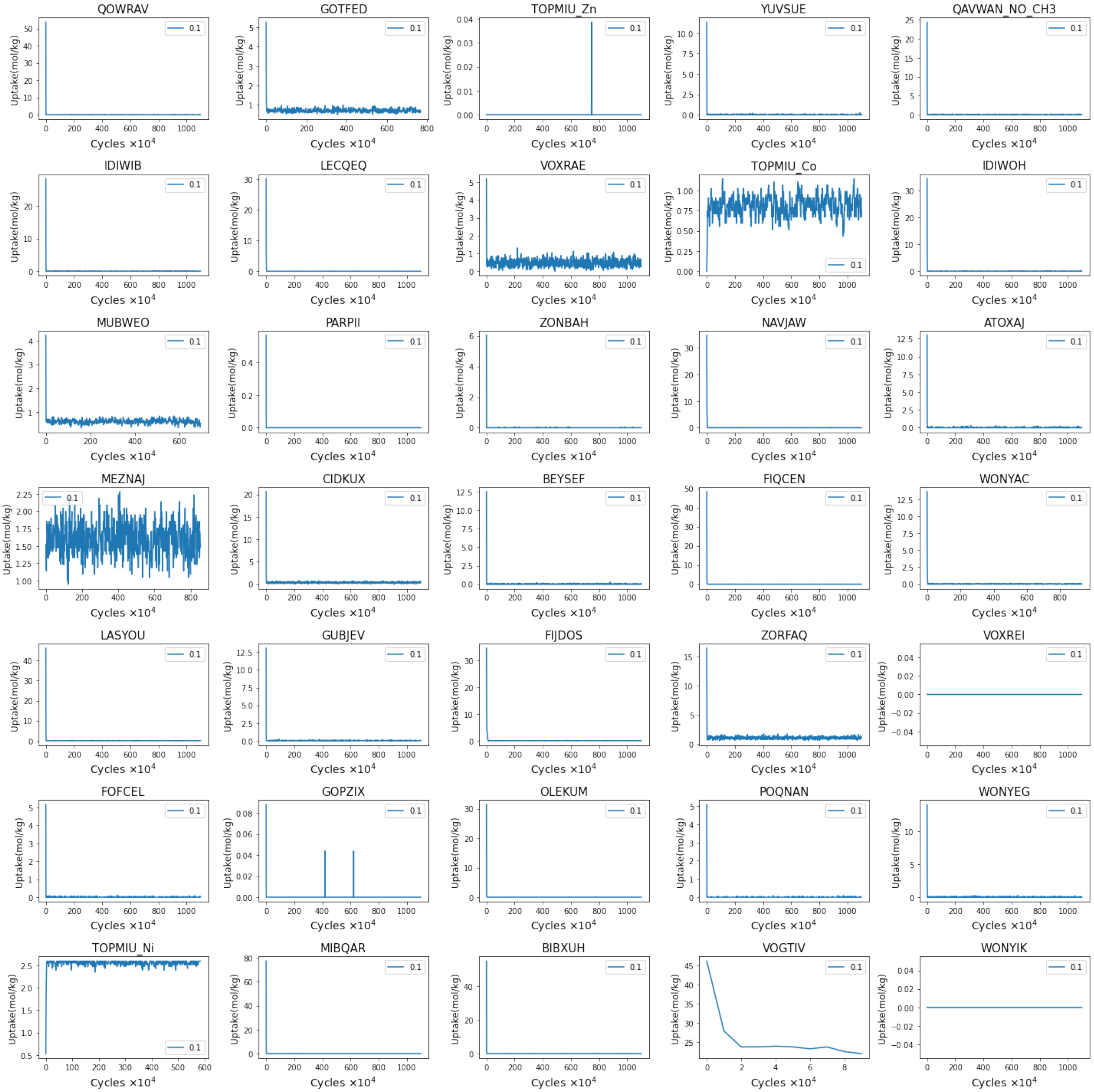

Convergence plots for all pristine MOFs at pressure point P/P_0 = 0.2 and 0.3

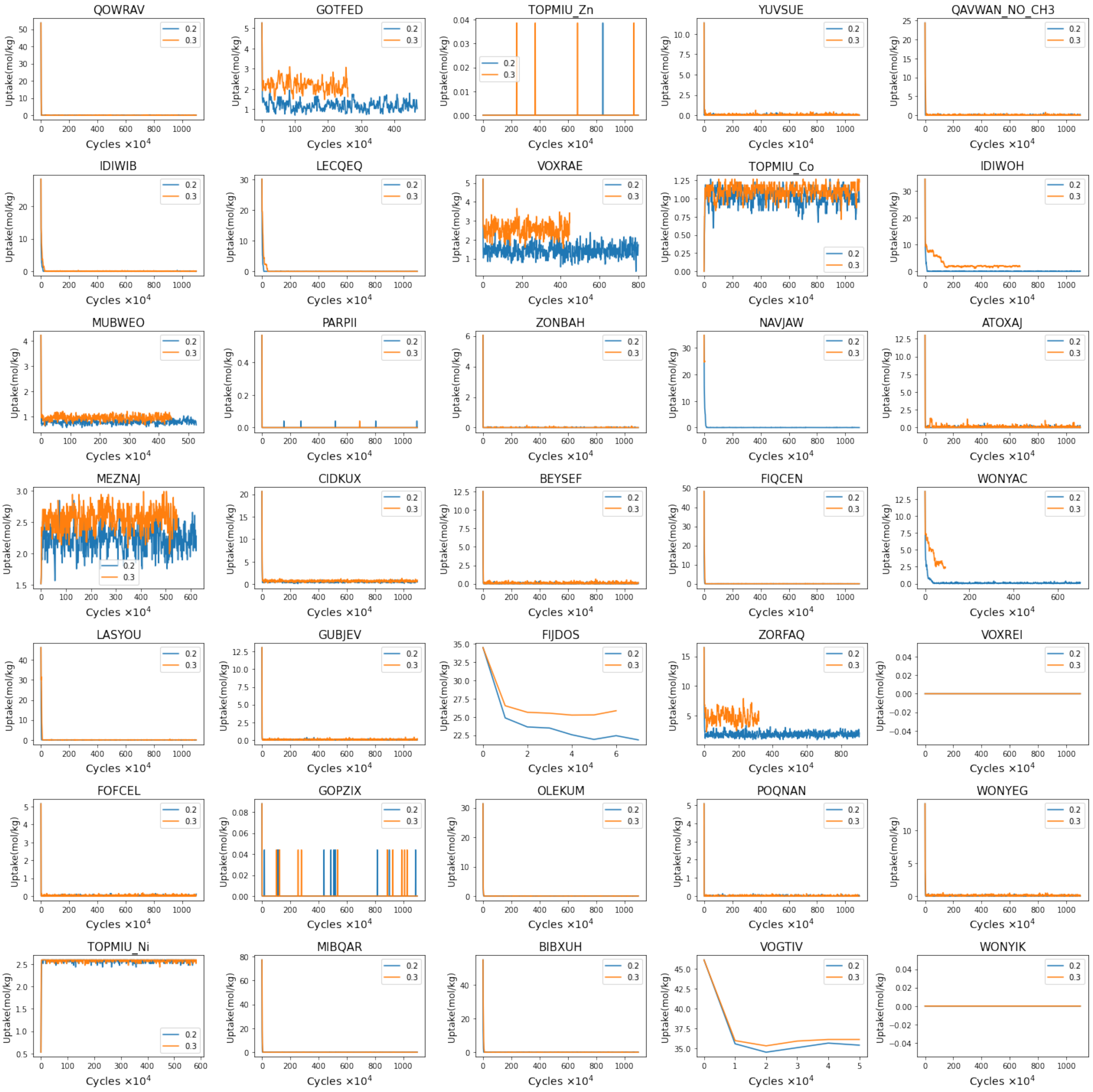

Convergence plots for all pristine MOFs at pressure point $P/P_0 = 0.5$

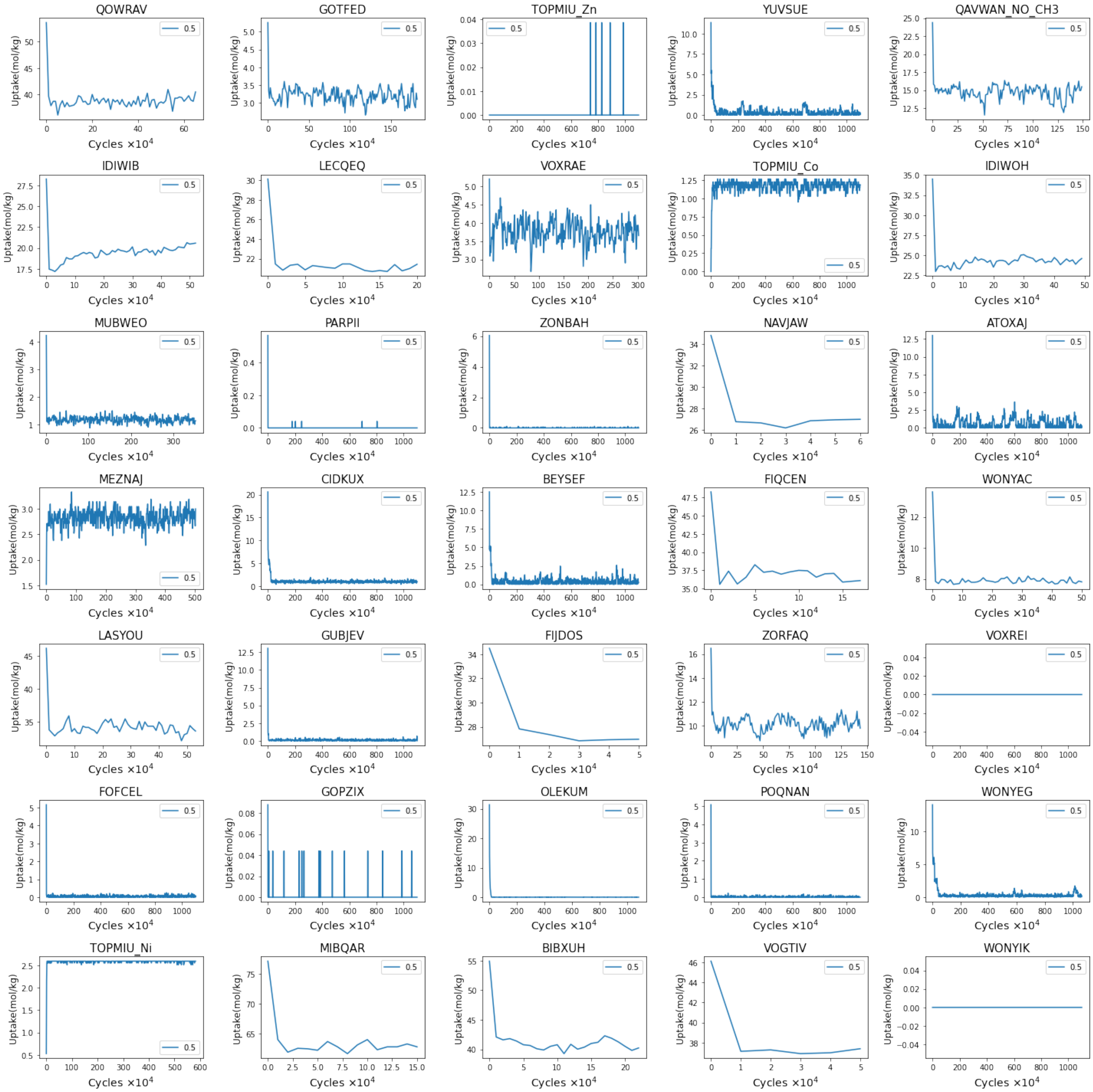

Convergence plots for all pristine MOFs at pressure point P/P0 = 0.7, 0.8 and 1.0

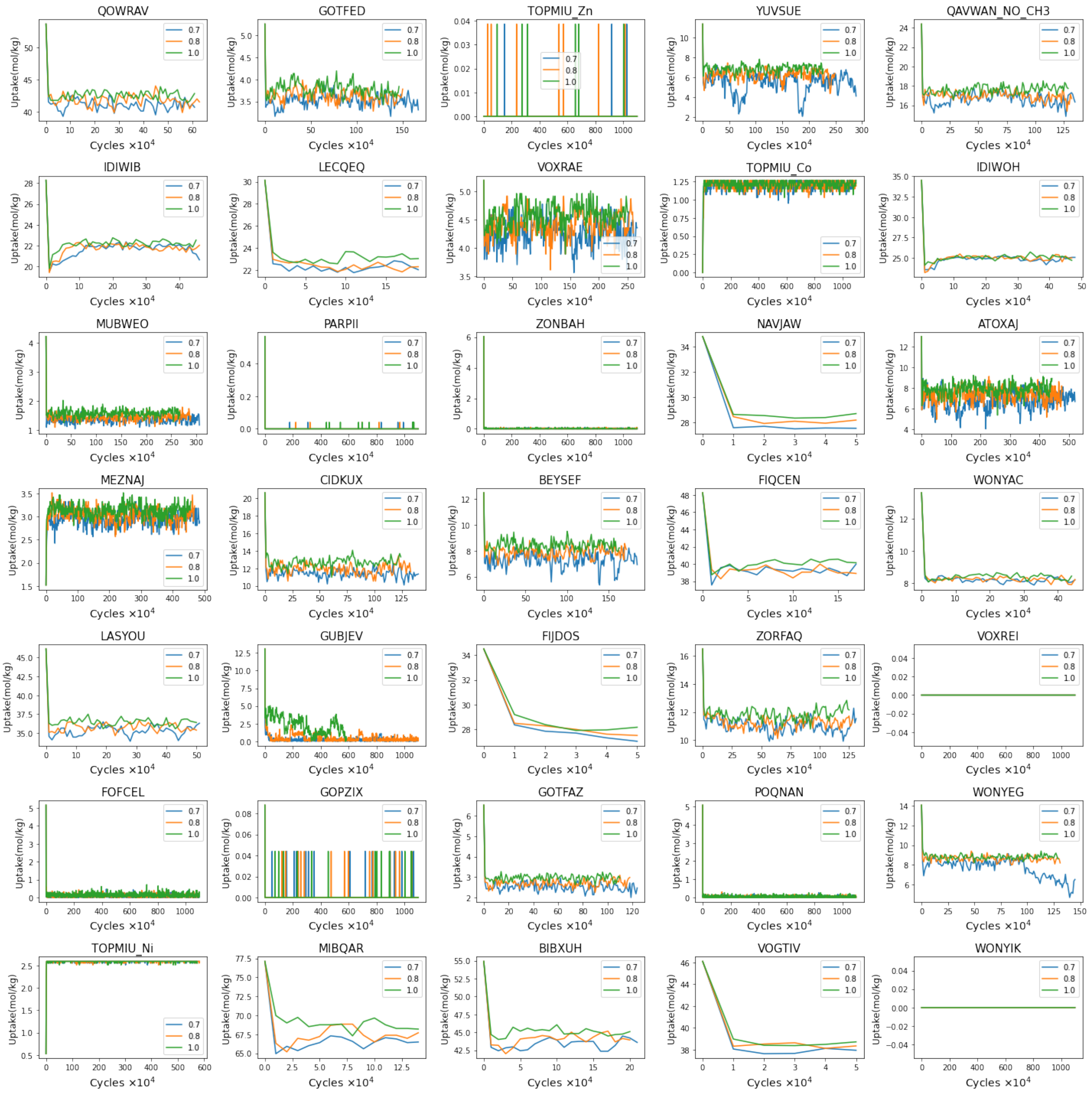

Figure S3: Convergence plots for GCMC simulations in pristine MOFs at 298 K

V. Convergence plots for GCMC simulations for defective MOFs

Convergence plots for all defective MOFs at pressure point $P/P_0 = 0.1$

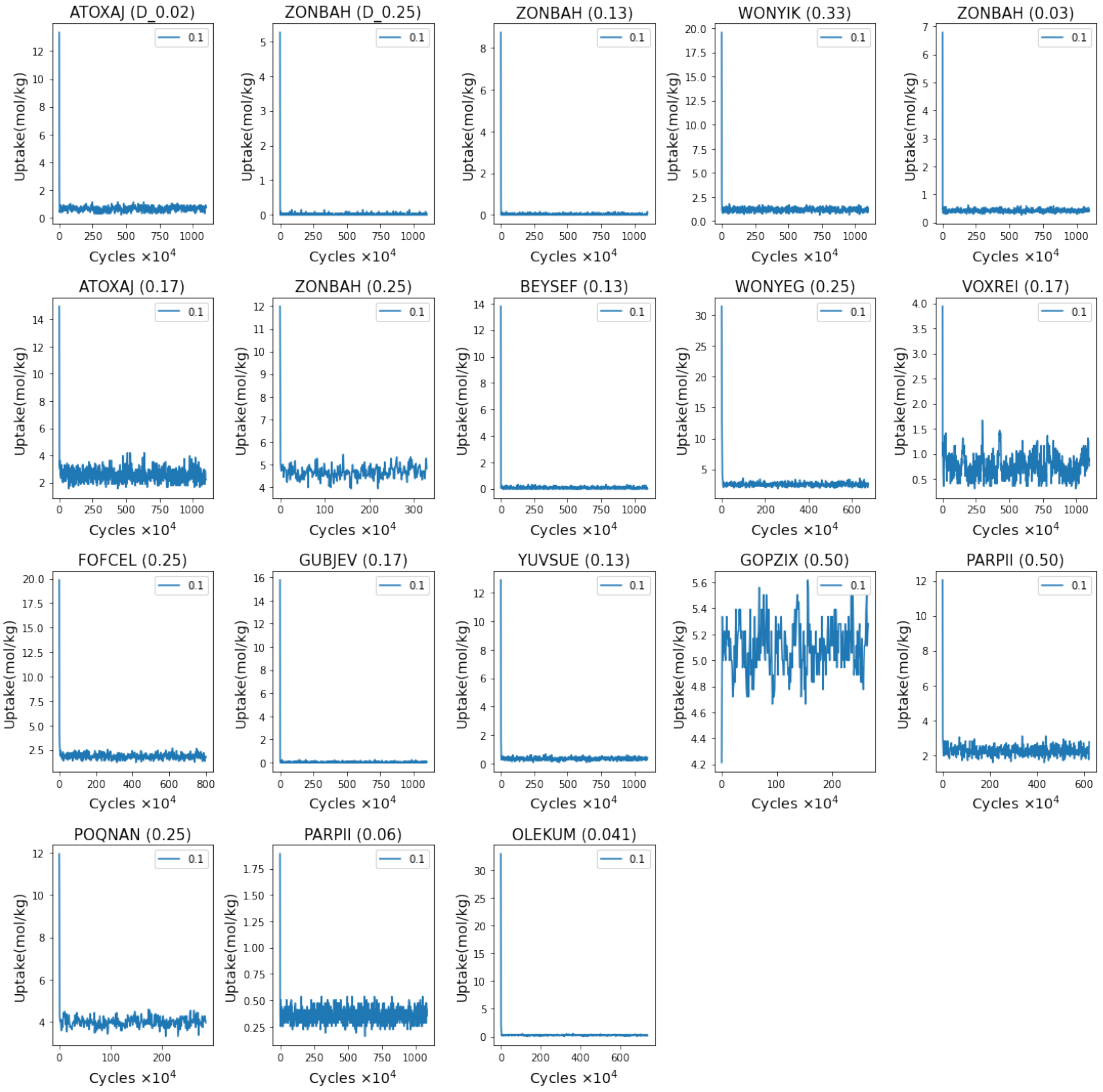

Convergence plots for all defective MOFs at pressure point P/P_0 = 0.2 and 0.3

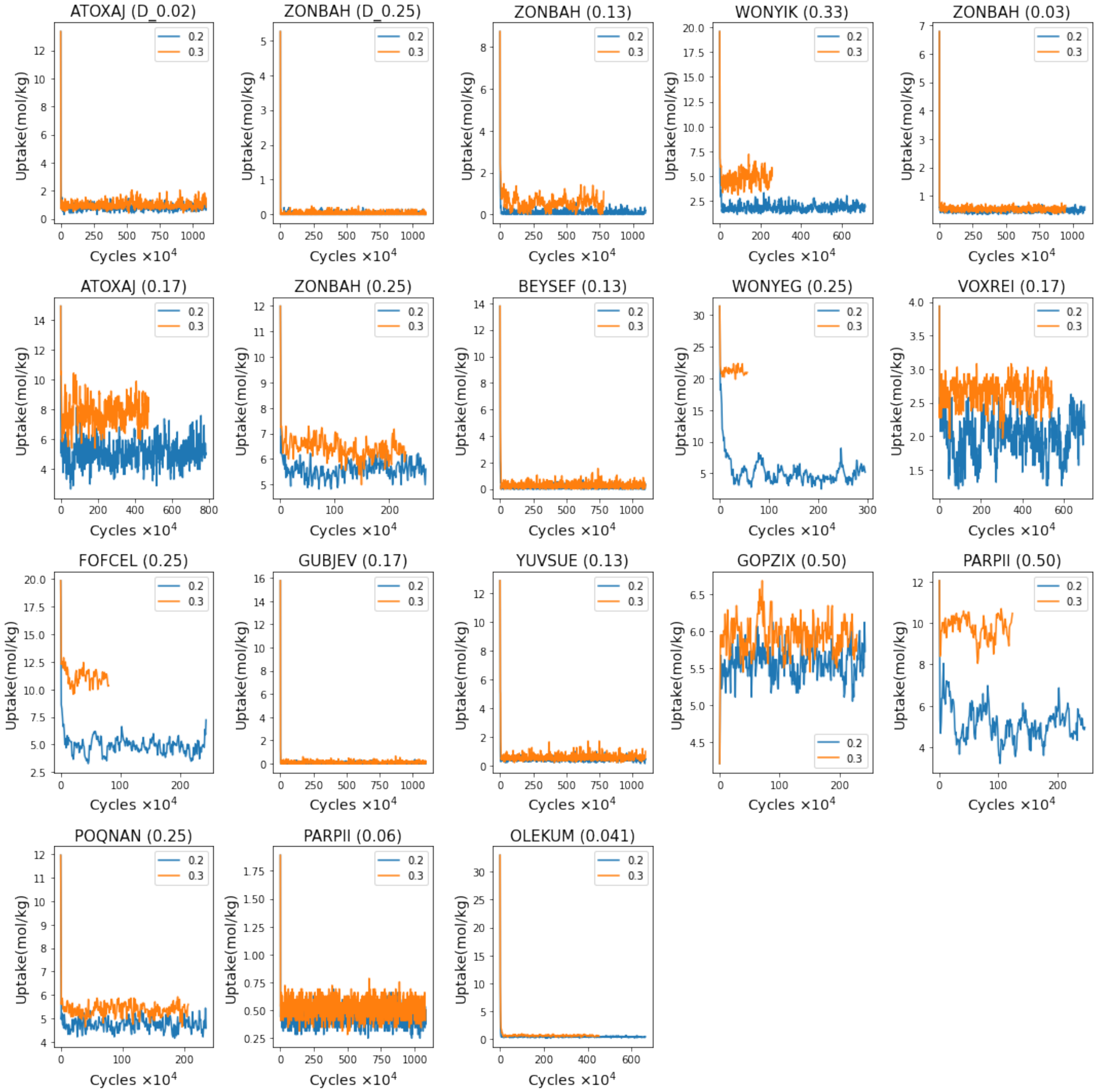

Convergence plots for all defective MOFs at pressure point $P/P_0 = 0.5$

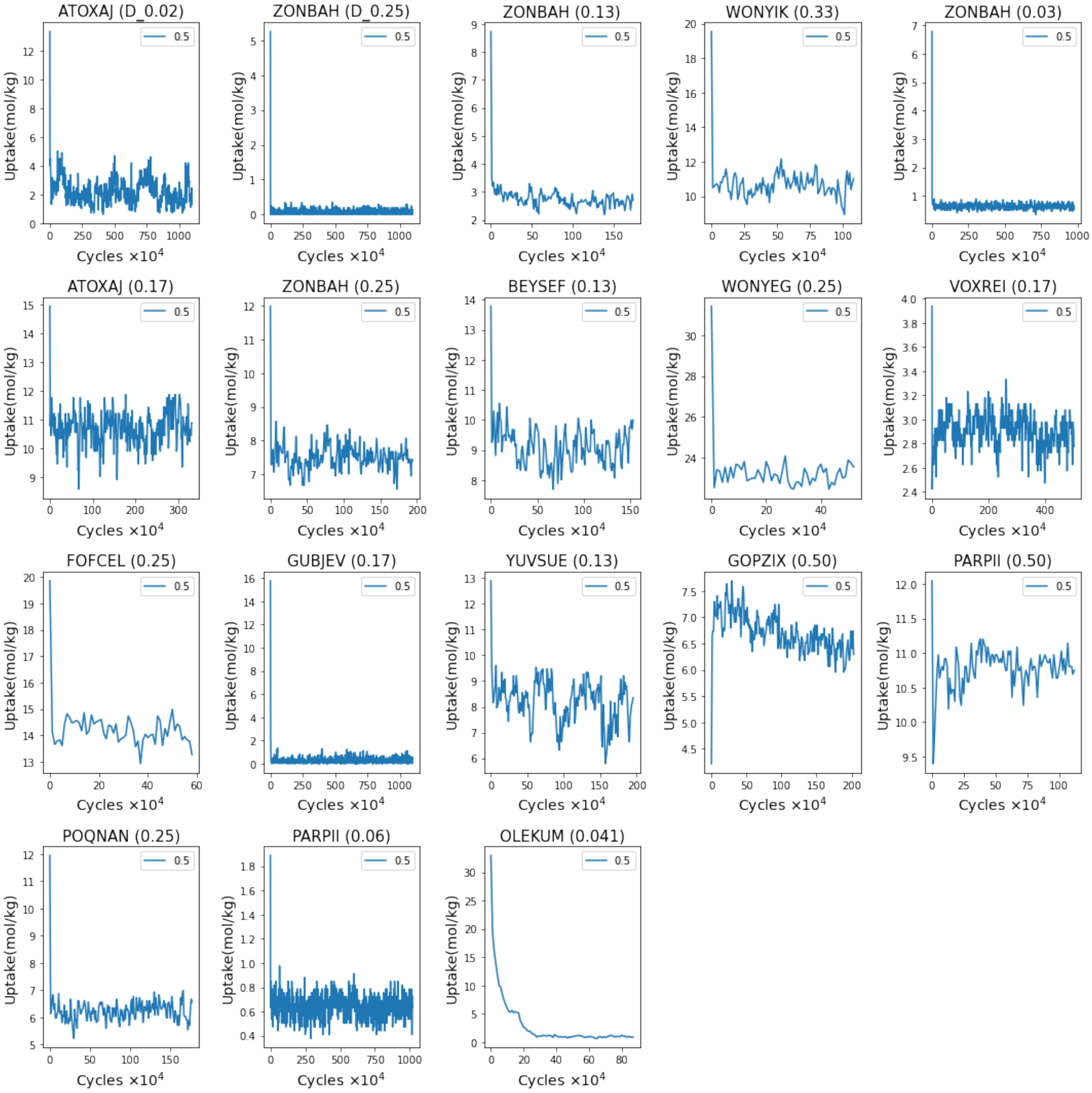

Convergence plots for all defective MOFs at pressure point P/P_0 = 0.7, 0.8 and 1.0

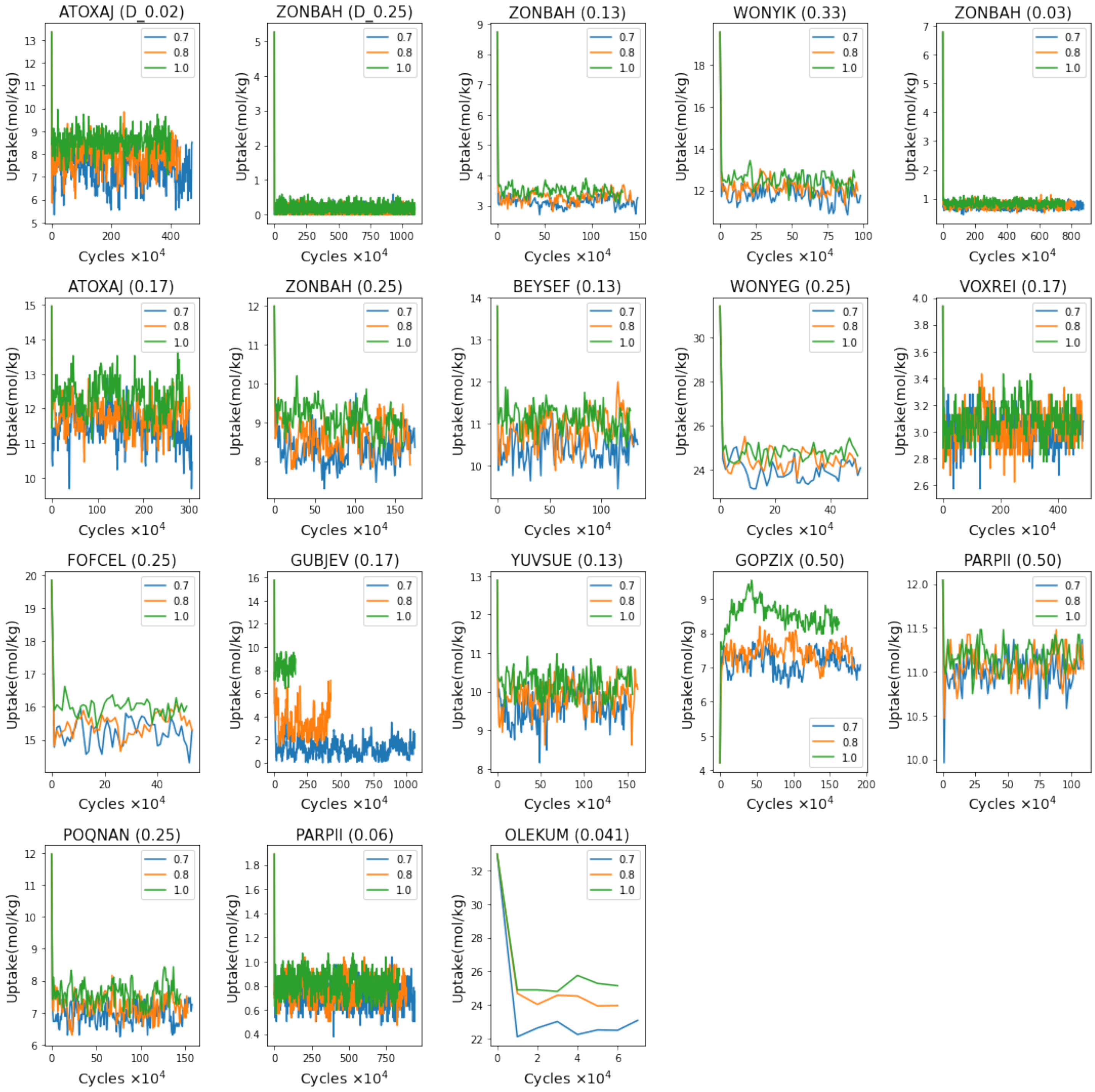

Figure S4: Convergence plots for GCMC simulations in defective MOFs at 298 K

References:

(1) Burtch, N. C.; Jasuja, H.; Walton, K. S. Water stability and adsorption in metal-organic frameworks. *Chem Rev* **2014**, *114* (20), 10575-10612, DOI: 10.1021/cr5002589.

(2) Batra, R.; Chen, C.; Evans, T. G.; Walton, K. S.; Ramprasad, R. Prediction of water stability of metal–organic frameworks using machine learning. *Nature Machine Intelligence* **2020**, *2* (11), 704-710, DOI: 10.1038/s42256-020-00249-z.
